# Dichloroacetate Improves Animal Survival, Growth, Neuromuscular Activity, Mitochondrial Stress and Physiology, and Elevated Lactate in *C. elegans* *pdha‐1* and *dld‐1* RNAi Models of Pyruvate Dehydrogenase Complex Deficiency (PDCD)

**DOI:** 10.1002/jimd.70257

**Published:** 2026-10-07

**Authors:** Cristina Remes, Neal D. Mathew, Victoria Miranda, Suraiya Haroon, Tyler O'Hara, Vernon E. Anderson, Manuela Lavorato, Kelsey Keith, Rui Xiao, Eiko Nakamaru‐Ogiso, Marni J. Falk

**Affiliations:** ^1^ Mitochondrial Medicine Frontier Program, Division of Genetic and Genomic Medicine, Department of Pediatrics The Children's Hospital of Philadelphia (CHOP) Philadelphia Pennsylvania USA; ^2^ Department of Pediatrics Perelman School of Medicine, University of Pennsylvania Philadelphia Pennsylvania USA; ^3^ Department of Biomedical and Health Informatics The Children's Hospital of Philadelphia Philadelphia Pennsylvania USA; ^4^ Department of Biostatistics, Perelman School of Medicine University of Pennsylvania Philadelphia Pennsylvania USA

**Keywords:** dichloroacetate (DCA), *DLD*, growth, mitochondria, neuromuscular activity, *PDHA1*, pyruvate dehydrogenase complex, RNA interference

## Abstract

Pyruvate dehydrogenase complex (PDHc) deficiency (PDCD) is a primary mitochondrial disorder characterized by neurodevelopmental disability, altered intermediary metabolism, and early mortality. Dichloroacetate (DCA), a pyruvate analog, activates PDHc and remains under clinical investigation for treatment of PDCD. Here, we studied the in vivo efficacy of a five‐point log concentration range of DCA on animal health and metabolism in *C. elegans* with feeding RNA interference (RNAi) expression knockdown of either *PDHA‐1* or *DLD‐1* homologs at graded degrees to model variable disease severity. These worm models recapitulate phenotypic features of PDCD observed in human patients, including reduced survival, delayed growth, locomotor impairment, and elevated lactate and/or pyruvate tissue levels. DCA treatment appeared well‐tolerated, with no gross morphologic toxicity seen at doses up to 25 mM. Significantly improved health, survival, tissue lactate levels, and mitochondrial physiology were observed at 25 mM in *pdha‐1(RNAi)* knockdown animals. DCA treatment in *dld‐1(RNAi)* *C. elegans* models (undiluted, 1:20 dilution, and 1:100 dilution) significantly improved survival, neuromuscular function, and metabolic phenotypes primarily in the moderate (1:20) and/or mild (1:100) *dld‐1(RNAi)* deficiency strains, but not in full‐dose *dld‐1(RNAi)*. Importantly, linear growth, neuromuscular activity, and mitochondrial physiology were significantly improved with DCA treatment even in the most severe *dld‐1(RNAi)* undiluted model. Overall, preclinical modeling provides objective evidence of DCA therapeutic efficacy in *C. elegans* knockdown strains for two well‐conserved homologs of *PDHA1* and *DLD* representing distinct genetic etiologies of PDCD, with demonstrated beneficial effects on survival, healthspan, tissue lactate, and mitochondrial physiology. These data further confirm that DCA's therapeutic effect correlates with PDHc disease phenotype severity in *dld‐1(RNAi)* animals.

## 1 Introduction

Pyruvate dehydrogenase complex (PDHc) deficiency (PDCD, OMIM 312170) is a rare genetic mitochondrial disorder, mainly affecting the central nervous system with manifestations of neurodevelopmental delay, structural brain abnormalities, neuromuscular disability, lactic acidosis, and increased plasma pyruvate [1, 2, 3, 4, 5, 6]. Human PDHc is a large (approximately 9 MDa), highly structured multi‐subunit enzyme complex [7, 8] located in the mitochondrial matrix that catalyzes the irreversible oxidative decarboxylation of pyruvate into acetyl‐CoA. Pathogenic variants in any of the genes encoding PDHc subunits can cause PDCD, where the most common occur in the gene encoding the pyruvate dehydrogenase E1α subunit (*PDHA1*, OMIM 300502) [9]. Less commonly, pathogenic variants of dihydrolipoamide dehydrogenase (*DLD*, OMIM 246900) cause 6% of PDCD cases [2]. In addition to its role in PDHc activity, DLD also serves as the dihydrolipoamide dehydrogenase (E3 subunit) in four other keto acid dehydrogenase complexes [10, 11, 12]. Sequence alignment of human and *C. elegans* PDHA1 and DLD shows a high degree of protein sequence homology, with 58.7% and 68.4% sequence identity and 96.7% and 96.9% sequence similarity, respectively (Figures S1A and S8A).

Currently, no US Food and Drug Administration (FDA)‐approved treatments or cures exist for primary PDCD. However, beneficial effects of dichloroacetate (DCA) have been reported in patient case reports [13, 14], animal models [11], and cellular models [15, 16, 17, 18]. Two mechanisms by which DCA increases PDHc activity have been proposed. The first mechanism involves inhibition of PDHc kinases, thereby keeping PDHc in its unphosphorylated, catalytically active state [19, 20]. Another possible mechanism that explains DCA's beneficial effects in PDCD that has been explored in human fibroblasts is stabilization of the PDHc enzymatic complex to reduce its rate of degradation [15, 21]. However, cellular models have been shown to have selective sensitivity to DCA [21], the reason for which is currently not fully understood.

A multisite, Phase 3, randomized, placebo‐controlled, cross‐over clinical trial investigating the therapeutic effect of DCA in human PDCD subjects has been completed, with regulatory review pending from the FDA [22]. However, due to the inherent rare nature of this disease, it is not feasible to complete a second adequate and well‐controlled clinical trial of DCA effect in PDCD. Therefore, we sought to provide confirmatory, objective, preclinical evidence of dose‐dependent safety and therapeutic efficacy of DCA as a treatment for PDCD. To this end, we used two *C. elegans* RNA interference (RNAi) knockdown strains (*pdha‐1* and *dld‐1*) to model PDCD [11, 23] and evaluate the efficacy of DCA on animal survival, health, and diverse aspects of mitochondrial physiology. In addition, we used a graded RNAi feeding approach to characterize the *dld‐1 (RNAi)* knockdown models at three different dilutions to evaluate DCA treatment effects in models with different degrees of DLD deficiency and phenotypic severity at the level of animal survival, linear growth, neuromuscular function, and intermediary metabolic function, including tissue lactate and pyruvate levels and mitochondrial physiology assessments.

## 2 Methods

An overview of the experimental design and conditions is provided in Table S2.

### 2.1 *C. elegans* Strains and Maintenance

The original N2 Bristol, GL347 (*hsp‐6p::GFP*), VS21 (*myo‐2p::mCherry*), and the SJ4103 *C. elegans* strain carrying the zcls14 construct (*myo‐3p::GFP(mit)*) were obtained from the *Caenorhabditis* Genetics Center (CGC) at the University of Minnesota. The GL347 (*hsp‐6p::GFP*) and VS21 (*myo‐2p::mCherry*) strains were crossed to generate a wild‐type line expressing both *hsp‐6p::GFP* and *myo‐2p::mCherry*. Worms were maintained under standard conditions at 20°C on nematode growth medium (NGM) seeded with *Escherichia coli* OP50, unless stated otherwise. DCA was provided under a sponsored research agreement from Saol Therapeutics to evaluate the potential efficacy and tolerability of a human‐grade formulation used in PDCD patient clinical trials, as opposed to a prior study by this research group of commercially available laboratory‐grade DCA [11].

### 2.2 Synchronization of *C. elegans*

Embryos were obtained from gravid adult animals by exposure to commercial concentrated germicidal bleach containing 8.25% sodium hypochlorite (Clorox). Worms were collected from NGM plates with 4 mL S. Basal (23.4 g NaCl, 4 g K_2_HPO_4_, 25 g KH_2_PO_4_, 4 mL cholesterol [5 mg/mL in ethanol], and 4 L Milli‐Q water, pH = 7). A total of 1 mL fresh bleach and 500 μL 5 N NaOH were added to the worm suspension, and the tubes were mixed for approximately 2 min, until no visible worm bodies were present. The samples were centrifuged at 199 × *g* for 1 min, and the supernatant was immediately removed. The pellet was subsequently washed twice with 10 mL S. Basal followed by centrifugation at 1200 × *g* for 1 min.

### 2.3 RNAi Feeding Knockdown of PDHA‐1 and DLD‐1 in *C. elegans*

Knockdown of PDHA‐1 and DLD‐1 in *C. elegans* was achieved by feeding transfected *E. coli* . *C. elegans* embryos were grown on NGM supplemented with 0.2 mM isopropyl β‐d‐1‐thiogalactoside (IPTG), 100 μg/mL ampicillin, treated with DCA or water, and seeded with *pdha‐1(RNAi)* (clone T05H10.6, *C. elegans* ORF Collection, Horizon), *dld‐1(RNAi)* (clone LLC1.3, Ahringer RNAi collection, Source BioScience) or control RNAi (clone L4440, Ahringer RNAi collection, Source BioScience). Prior to the knockdown experiments, all clones were sequence‐verified. For most experiments the *dld‐1(RNAi)* was undiluted or diluted with L4440 at 1:20 (*dld‐1(1:20 RNAi)*) or 1:100 (*dld‐1(1:100 RNAi)*). Unless stated otherwise, worms were analyzed at the L4 + 1 day.

### 2.4 *C. elegans* Gross Morphology Analysis

Wild‐type (N2 Bristol) DLD‐1 and PDHA‐1 knockdown worms were grown on solid media treated or untreated with DCA and observed at L4 + 5 days of adulthood to visually identify toxicity at the level of gross morphologic toxicity and identify the maximal safe effective dosage exposure. Worms were anesthetized by incubation with 0.5 mM sodium azide for 15 min at room temperature and imaged with a ZEISS Stemi 305 microscope.

### 2.5 *C. elegans* Lifespan Analyses Using WormScan

Wild‐type (N2 Bristol), DLD‐1 and PDHA‐1 knockdown worms, treated with DCA or water from embryo phase, were age‐synchronized by bleaching as described above. Stage L4 animals were then transferred onto 6 cm plates (30–60 worms per plate) containing 50 μM 5‐fluorodeoxyuridine (FUDR), 0.2 mM IPTG, 100 μg/mL ampicillin, and seeded with the corresponding RNAi bacteria, and treated with DCA or water. Two consecutive images (one image before and one image after light stimulation) were recorded every 48 h using an Epson V800 scanner. Worm survival was scored using WormScan Machine Learning (ML) v6.3, a Python desktop application developed in‐house. The images are compared using two deep learning models. A YOLO26 object detector [24, 25] localized individual worms in each scanned image, and a MobileNetV2 classifier [26] determined alive/dead status from a stitched side‐by‐side composite of the two time points. Each image pair was loaded into the application, the plate area was isolated using a circular crop, and any annotations or writing on the plate were masked. The YOLO26 detector (trained on 18 plates, 417 annotations, validation mAP50 = 0.91) identified worm locations in the T1 image. For each detection, a 256 × 128 pixel composite was generated by stitching the same region from both T0 and T1 time points, which was passed to the MobileNetV2 classifier (trained on 1425 alive and 251 dead examples; balanced validation accuracy = 98%) for alive/dead assignment. Detections with confidence below 0.25 (detector) or 0.50 (classifier) were excluded. Counts were exported for survival curve calculation, with manual correction available for any miscalled worms. The image analysis tool WormScan ML v6.3.1 (https://doi.org/10.5281/zenodo.21042471) is archived on Zenodo [27].

### 2.6 Manual *C. elegans* Lifespan Analyses

Wild type (N2 Bristol), *pdha‐1 (RNAi)*, and *dld‐1 (RNAi)* treated with 25 mM DCA or water from embryo phase were age‐synchronized by bleaching. At the L4 stage, approximately 30 worms were transferred to 3.5 cm NGM plates supplemented with 0.2 mM IPTG and ampicillin 100 μg/mL, and seeded with the corresponding RNAi bacteria. Worms were scored daily for survival and transferred to fresh plates until reproduction ceased. Animals unresponsive to gentle stimulation with a wire pick were scored as dead. Animals lost due to nonnatural causes were censored from survival analysis. Three biological replicates were analyzed for all conditions.

### 2.7 *C. elegans* Light Stimulated Locomotor Activity Measurements

Automated locomotor activity analysis was conducted in 24‐well plates prepared with transparent GelRite‐based media to enable optimal imaging, using a previously described method [28]. Each well contained 1 mL of media (8 g/L GelRite, 3 g/L NaCl, 2.5 g/L peptone, 40 μM FUDR, and 100 μg/mL ampicillin) supplemented with either 0.2 or 2 mM IPTG for *dld‐1* and *pdha‐1* RNAi conditions, respectively. Media between wells consisted of 6.7 g/L GelRite and 3 g/L NaCl. Plates were treated with the appropriate concentration of DCA or water (buffer control), seeded with the corresponding RNAi bacteria, and allowed to dry overnight before use. Wild‐type (N2 Bristol), *dld‐1 (full‐dose RNAi*), and *pdha‐1 (RNAi)* knockdown worms were age‐synchronized by bleaching onto the appropriate 10 cm RNAi plates, as described above. L4‐stage worms were collected with 10 mL S. Basal and concentrated to 2 worms/μL. A total of 10 μL of worm suspension (20 worms) was added to each well, and the plates were allowed to dry for 15 min. Six technical replicates were included per condition on each plate. Before imaging, plate lids were treated with anti‐fog spray to prevent condensation. Plates were imaged twice daily, before and after blue‐light stimulation, using the WormWatcher robot over a 30‐day period, as previously described [28]. For each experimental plate, locomotor activity was normalized to the corresponding wild‐type control measured on the same plate in an effort to account for variability between plates. Normalized values were then used for statistical analysis and visualization. Data were analyzed and visualized using R [29].

### 2.8 *C. elegans* Linear Growth (Length) Measurements

Wild‐type (N2 Bristol), PDHA‐1, and DLD‐1 knockdown worms were imaged on solid media using an Epson V800 scanner, and the length of each individual worm was analyzed using ImageJ. Three biological replicates per condition were performed, with a total of 30 worms per condition. The lengths of the DLD‐1 and PDHA‐1 knockdown worms were normalized to the length of the wild‐type worms.

### 2.9 Neuromuscular Activity and Locomotor Behavior Analyses in *C. elegans*

Motility of wild‐type (N2 Bristol), PDHA‐1 and DLD‐1 knockdown worms in liquid media was measured using a thrashing assay. Worms were placed in S. Basal, and a 10 s video was collected using a Basler USB Camera (model #108014) with a KOWA industrial lens 75 mm/F2.5. The FIJI plugin wrMTrck [30] was used to determine the body bends/s of each individual *C. elegans* . Three biological replicate experiments were performed, with a total of 60 worms analyzed for each condition.

### 2.10 Mitochondrial Unfolded Protein Response (UPR^mt^ ) Induction Analysis

The *C. elegans* N2 (*hsp‐6p::gfp* + *myo‐2p::mCherry*) strain was used to quantify induction of the UPR^mt^ [31]. Wild‐type, DLD‐1, and PDHA‐1 knockdown worms were grown from the embryo stage on 10 cm NGM plates containing 0.2 mM IPTG, 100 μg/mL ampicillin, treated with DCA or buffer control, and seeded with the corresponding RNAi bacteria. Day 1 adult worms were washed in S. Basal to a density of 1 worm/μL. The worm suspension was then dispensed into a 384‐well plate (Optically Clear Polymer Bottom, USA Scientific #5678‐1091) at a volume of 50 μL/well. Subsequently, 50 μL of sodium azide (NaN_3_) (0.1 M supplemented with 0.01% Tween‐20) was added to each well to anesthetize the worms for imaging and incubated for 15 min at room temperature. Mitochondrial stress was then measured using a CellInsight CX5 High Content Screening system (ThermoFisher Scientific). The red channel was used to identify and quantify the number of worms in each well, and the total fluorescence detected in the green channel was used to quantify the total UPR^mt^ of each well. The total fluorescence measured in the green channel was then normalized to the number of animals to obtain the UPR^mt^ per worm. Three biological replicates were performed, with eight technical replicates per condition.

### 2.11 Mitochondrial Membrane Potential Relative Analyses by Flow Cytometry

N2 Bristol worms were grown from the embryo phase on 10 cm NGM plates supplemented with 2 mM IPTG and 100 μg/mL ampicillin, seeded with *pdha‐1(RNAi)* or L4440 control bacteria until Stage L4. Worms were collected with 10 mL S. Basal and transferred onto 6 cm RNAi plates supplemented with 1 μM TMRE and 4 μM MTG for 24 h at 20°C. Worms were subsequently washed twice with 10 mL S. Basal and transferred to RNAi plates for 1 h at 20°C to allow intestinal clearing of the fluorescent dyes, and imaged with a CellInsight CX5 high‐content imager or analyzed with flow cytometry using a BioSorter (Union Biometrica). For BioSorter analyses, the following excitation wavelengths were used: *λ*_excitation_ = 488 nm and *λ*_emission_ = 510 nm. The fluorescence intensity in the red and green channels was normalized by the size (time of flight) of the worms (Figure S5). To determine the normalized membrane potential, the fluorescence measured in the red channel was normalized to the fluorescence measured in the green channel.

### 2.12 *C. elegans* Extracellular Flux Analysis

Baseline and maximal oxygen consumption rates (OCRs) were assessed using a Seahorse XF Pro Analyzer (Agilent Technologies) in an adaptation of a published protocol [32]. Twenty‐four hours prior to the experiment, the XF Pro heater was turned off and the sensors hydrated with 200 μL of XF Calibrant at 37°C in a non‐CO_2_ incubator. Micropipette tips were washed with 0.1% Triton X‐100 (v/v) in S. basal to inhibit worms from sticking to the plastic. Wild‐type (N2 Bristol), *pdha‐1(RNAi)*, and *dld‐1 (RNAi)* knockdown worms, treated with water or 25 mM DCA were collected at L4 stage, were washed two times with 1 mL S. basal, diluted with S. basal to 3 worms/10 μL, and transferred in 160 μL NaN_3_ (Sigma Aldrich S2002‐25G) dissolved in S. basal at 25 μM and 50 mM final concentrations upon worm exposure, respectively. The optimal concentration of carbonyl cyanide 4‐(trifluoromethoxy)phenylhydrazone (FCCP) was determined using a titration curve with wild‐type (N2 Bristol) (Figure S4B), *pdha‐1(RNAi)* (Figure S4C), and *dld‐1(RNAi)* (Figure S12C) worms. The assay timing was 2 min of mixing, 30 s delay, and 2 min of measurement, with cycles repeated four times for baseline, seven times for FCCP, and another four times for NaN_3_. Fifteen technical replicates were used per condition for each of three biologically independent experiments run at approximately 21°C–23°C. Non‐mitochondrial O_2_ consumption was not subtracted from baseline and maximal measures, as there was no significant difference between groups and the contribution was minimal (Figure S4E). Statistical analysis was performed in Prism 10.2.2 (GraphPad) on OCR values following normalization to worm count. The average OCR within a measurement period was determined for each well, with median response within a biological replicate taken across 15 technical replicates. Differences between wild‐type and knockdown worms and between untreated and DCA‐treated worms were analyzed by Welch's *t*‐test. Shown are mean ± standard error of the mean.

### 2.13 Lactate and Pyruvate Analyte Analyses

Wild‐type (N2 Bristol), *pdha‐1(RNAi)*, *dld‐1(RNAi)*, *dld‐1(1:20 RNAi)*, and *dld‐1(1:100 RNAi)* worms were grown on 15 cm NGM plates with 0.2 mM IPTG, 100 μg/mL ampicillin, treated with 25 mM DCA or water, from the embryo stage until Stage L4 + 1. Animals were then washed with 10 mL S. Basal and collected in 15 mL conical tubes. After washing three times with S. Basal, worms were flash‐frozen in liquid nitrogen and stored at −80°C. Lactate and pyruvate were spectrophotometrically analyzed as previously described [33].

### 2.14 Western Immunoblot Analyses

Wild‐type (N2 Bristol), PDHA‐1, and DLD‐1 knockdown worms were synchronized by bleaching. Stage L4 worms (10 worms per condition) were washed three times with S. Basal buffer and stored on ice. An equal volume of RIPA buffer and 6× Loading Dye (0.3 M Tris–HCl, 12% SDS, 0.06% bromophenol blue, 50% glycerol, and 0.6 M DTT) was subsequently added, and samples were incubated at 95°C for 15 min. Samples were then centrifuged at 21 300 × *g* for 20 min, and the supernatant was loaded onto Mini‐PROTEAN TGX Precast Protein Gels, 4%–15% (Bio‐Rad). Proteins were blotted onto polyvinyl difluoride membrane using Wet Transfer Buffer (38.6 mM glycine, 47.9 mM Tris, and 20% methanol, pH 8.3) at 4°C for 1 h at 350 mA. Membranes were first blocked in Intercept (TBS) Blocking Buffer (LICORbio) at room temperature for 1 h. The blocked membranes were incubated overnight with primary antibodies at the following dilutions: PDHA1 (Abcam, ab110334, 1:1000 dilution), DLD (Abcam, ab133551, 1:1000 dilution), beta‐actin (GeneTex, GTX109639, 1:1000 dilution), alpha‐tubulin (Sigma‐Aldrich, T9026, 1:1000 dilution). Membranes were then washed three times with 1× TBS‐Tween 20 (TBST; TBS with 0.1% Tween 20) for 10 min and incubated with secondary antibodies (IRDye 800CW Anti‐Rabbit IgG Goat and IRDye 680CW Anti‐Mouse IgG Goat) for 1 h at room temperature. Membranes were then washed at room temperature three times for 10 min with TBST and two times for 10 min with TBS, and imaged using an Odyssey CLx infrared imaging system. The relative protein band densities were quantified using ImageJ [34]. The full gel images for PDHA‐1 are in Figure S1B, and the full gel images for DLD‐1 are in Figure S8B.

### 2.15 Confocal Imaging

Confocal imaging was performed using the *C. elegans* SJ4103 strain carrying the zcls14 construct (*myo‐3p::GFP(mit)*), which expresses GFP in the mitochondria of the worm body‐wall muscle. The animals were grown in large numbers and bleached onto RNAi NGM plates prepared as described above, seeded with L4440 (control), *dld‐1 (RNAi)*, and *pdha‐1 (RNAi)* for 3 or 4 days, and treated with water or 25 mM DCA. On the day of imaging, the animals were collected and placed on a 3% agarose gel pad on a slide in 20–25 μM levamisole dissolved in S‐basal. The cover slip was placed on top of the paralyzed worms and sealed to the agarose pad with nail polish. The mitochondria in the body‐wall muscle were imaged with excitation at 488 nm and an emission range of 493–589 nm at 63× magnification on a confocal microscope to assess mitochondrial morphology. Mitochondrial morphology was evaluated both qualitatively and quantitatively. Qualitative assessment was based on the overall organization of the mitochondrial network, including the presence of elongated/tubular mitochondria, branching, fragmentation, and punctate or disorganized mitochondrial structures. For quantitative analysis, images were processed using Fiji/ImageJ software. Standardized image‐processing and thresholding parameters were applied across all experimental groups. The resulting binary mitochondrial masks were used for morphometric analysis. The following mitochondrial parameters were quantified: roundness, mitochondrial profile area, and average branch length. Roundness was used as a measure of mitochondrial shape, with higher values indicating a more rounded morphology. Mitochondrial profile area was calculated from individual segmented mitochondrial structures, whereas average branch length was determined from the skeletonized mitochondrial network. Skeletonization and branch‐length measurements were performed using the Skeletonize3D and AnalyzeSkeleton plugins in Fiji/ImageJ, adapting previously described approaches [35, 36, 37, 38]. Roundness and mitochondrial profile area were measured on individual mitochondrial structures (roundness: wild type, *n* = 1197; *dld‐1(RNAi)*, *n* = 1404; *pdha‐1(RNAi)*, *n* = 1192; *dld‐1(RNAi)* + 25 mM DCA, *n* = 591; *pdha‐1(RNAi)* + 25 mM DCA, *n* = 359; profile area: *n* = 1196, 1403, 1191, 591, and 359, respectively), and average branch length was measured on individual branches of the skeletonized network (*n* = 1778, 2941, 2140, 859, and 537, respectively). All measurements within a condition were pooled for analysis.

### 2.16 Statistical Analyses

Statistical analyses were performed using GraphPad Prism version 10.2.2. Unless otherwise noted, differences between wild‐type and knockdown worms were analyzed by Welch's *t*‐test, while comparisons of untreated knockdown worms and the dose–response to DCA were analyzed by one‐way ANOVA followed by post hoc analysis for a log‐linear regression trend if the ANOVA *p* value was significant. Statistical significance is reported for the ANOVA along with the estimated slope ± standard error (SE) of the slope. Data for individual conditions are presented as mean ± standard deviation (SD). For studies of survival, Kaplan–Meier survival curves were plotted and compared with a log‐rank Mantel–Cox test. Statistical significance was set at *p* value < 0.05, with a Bonferroni correction applied to account for multiple testing when needed. For the automated light‐stimulated locomotor activity, comparisons between groups were analyzed using ANOVA with a post hoc Tukey test. An adjusted probability (*p*) value of less than 0.05 was considered statistically significant. Plots were made using ggplot2 [29]. For mitochondrial morphology analyses, statistical analyses were performed using GraphPad Prism version 10.2.2, and comparisons between experimental groups were performed using one‐way ANOVA followed by Šídák's multiple comparisons test.

## 3 Results

### 3.1 DCA Treatment Significantly Rescued Disease Phenotypes in *pdha‐1(RNAi)* Worms

The most common cause (~77%) of PDCD is pathogenic variants in the PDHc alpha subunit gene (*PDHA1*) [9]. Therefore, we first characterized the effect of knocking down the ortholog, *pdha‐1*, in *C. elegans* by feeding wild‐type (N2 Bristol) worms with RNAi bacteria. We confirmed a 70% reduction of PDHA‐1 protein in the *pdha‐1(RNAi)* knockdown worms using western immunoblotting (Figure 1A,B and Figure S1B). Having established a *C. elegans* model for PDHA‐1 deficiency, similar to that developed previously [23], we investigated the effect of human clinical trial‐grade DCA on their disease phenotypes. Wild‐type and *pdha‐1(RNAi)* worms were treated from embryo phase on solid media supplemented with a five‐point log concentration dosing range of DCA (10 μM, 100 μM, 1 mM, 5 mM, and 25 mM); animals were subsequently observed at L4 + 1 and L4 + 5 days of adulthood to visually identify toxicity at the level of gross morphologic alterations and determine the maximal safe effective dosage exposure (Figures S2 and S3). No toxic effects on gross morphology were observed in the tested DCA dosage range.

**FIGURE 1:**
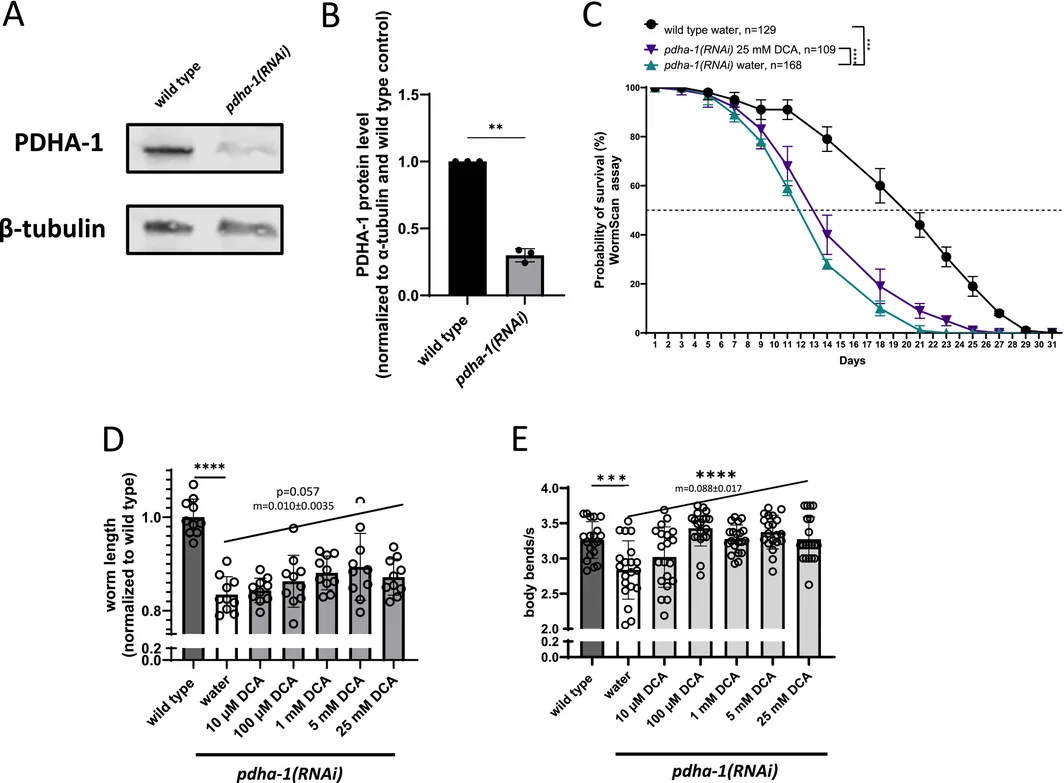
DCA treatment effect on overall animal health of *pdha‐1(RNAi)* knockdown worms. (A) Western blot analysis of PDHA‐1 protein levels after feeding N2 (Bristol) worms with *pdha‐1* or empty vector *RNAi* bacteria. A representative blot of *n* = 3 biological replicates is shown. (B) Quantification of the PDHA‐1 protein band density, confirming the specific knockdown of PDHA‐1 by feeding worms with *pdha‐1(RNAi)*. PDHA‐1 protein levels relative to β‐tubulin were normalized to wild type. Bars represent the mean ± SD of three biological replicates (**p < 0.01) . (C) Lifespan analysis of the *pdha‐1(RNAi)* knockdown worms (teal curve) in the presence of FUDR shows a significant decrease in lifespan relative to wild type (N2 Bristol, black curve, *p* = 0.0083), while treatment with 25 mM DCA significantly increased worm survival (purple curve, *p* < 0.0001). Three biological replicates are presented. (D) Worm growth analysis of the *pdha‐1(RNAi)* knockdown worms (white bars) show a ~18% decrease in length compared to wild‐type worms (N2 Bristol, dark gray bars, *p* < 0.0001), while treatment with 10 μM to 25 mM DCA (light gray bars) leads to a strong dose‐dependent increasing trend in worm length (one‐way ANOVA, *p* = 0.057, post hoc log linear trend shown). Data represent the mean ± SD of 10 individual animals. (E) *pdha‐1(RNAi)* knockdown worms (white bars) had reduced swimming (neuromuscular) activity relative to wild‐type control worms (N2 Bristol, dark gray bar, ***p < 0.001), which was significantly rescued by treatment with 10 μM to 25 mM DCA (light gray bars, one‐way ANOVA, ****p < 0.0001, log linear trend shown). Treatment with 100 μM, 1 mM, 5 mM, and 25 mM DCA lead to a recovery not significantly different than wild‐type swimming activity. Data represent the mean value ± SD of 20 individual animals.

Lifespan of the *pdha‐1(RNAi)* worms was first quantified by our previously established WormScan method performed in the presence of FUDR [39], where we observed a significant reduction in survival (*p* = 0.0083) relative to wild‐type control (N2 Bristol) worms (Figure 1C). Treatment with 25 mM DCA significantly increased the lifespan of the *pdha‐1(RNAi)* worms, with the median lifetime increased from 12 to 13.5 days, *p* < 0.0001 (Figure 1C). No effect was observed on the lifespan of the wild‐type worms after treatment with 25 mM DCA (Figure S1C). Furthermore, the effect of DCA on *pdha‐1(RNAi)* worms was independently confirmed using a standard manual lifespan assay without FUDR (Figure S1D), in which treatment with 25 mM DCA significantly increased lifespan compared to untreated *pdha‐1(RNAi)* worms (*p* = 0.013). In addition, worm growth was quantified in *pdha‐1(RNAi)* knockdown worms, which had an ~18% decrease in worm length compared to wild‐type animals (Figure 1D). The short growth phenotype showed a trend of rescue by DCA; one‐way ANOVA *p* = 0.057, with a linear slope of 0.010 ± 0.003 (Figure 1D). Post hoc analysis showed a significant difference in linear length between untreated *pdha‐1(RNAi)* animals and those treated with 5 mM DCA (*p* = 0.0369). The linear trend detection of the ANOVA analysis indicated the slope was ~3× its standard error (0.010–0.0035), which has been further confirmed by log‐linear regression of normalized worm length to log[DCA], where the slope is 0.012 ± 0.004 (Figure S1F).

Since PDHc deficiencies cause neuromuscular disease in human patients [2], we analyzed the neuromotor activity of the *pdha‐1(RNAi)* worms at the Day 1 adult stage using a swim‐locomotor (animal thrashing) assay of worms placed in liquid media [30]. Indeed, knocking down PDHA‐1 significantly decreased worm activity, *p* < 0.001. Treatments with DCA from 100 μM to 25 mM each significantly improved *pdha‐1(RNAi)* worm swim activity by one‐way ANOVA, *p* < 0.0001, with a linear response slope of 0.088 ± 0.017 (Figure 1E). Importantly, an activity level not significantly different from wild type was observed with all treatments above 100 μM DCA. In addition, we measured worm light‐stimulated activity on solid media, as previously described [28]. *pdha‐1(RNAi)* worms exhibited reduced activity compared to the wild‐type control, which was not altered by treatment with DCA at any tested concentration across six technical replicates (Figure S1E). In contrast, DCA treatment increased locomotor activity in wild‐type animals (Figure S1E).

We also sought to determine if treatment with DCA improved mitochondrial health at the level of integrated UPR^mt^ induction in *pdha‐1(RNAi)* worms. UPR^mt^ has been extensively characterized in *C. elegans* , where it triggers the upregulation of the mitochondrial chaperones *hsp‐6* (orthologue to human HSP70) and *hsp‐60* (orthologue to human HSP60, or HSPD1) to promote protein folding and maintain mitochondrial health. UPR^mt^ stress has been shown to be triggered by perturbations in mitochondrial physiology [40, 41, 42]. Therefore, activation of the *hsp‐6p* reporter was used as a quantitative indicator of mitochondrial dysfunction [43, 44]. For these assays, we used an N2 Bristol strain carrying an *hsp‐6p::gfp* reporter (green fluorescence) stably crossed to a *myo‐2p::mCherry* reporter line (red fluorescence) to quantify the degree of UPR^mt^ stress induction normalized per worm (Figure S4A). The *pdha‐1(RNAi)* worms had significantly increased mitochondrial stress induction relative to wild‐type animals, which DCA treatment significantly reduced in all tested dose conditions (Figure 2A). The greatest degree of UPR^mt^ stress reduction (86%, *p* = 0.0012) was observed after treatment with 25 mM DCA.

**FIGURE 2:**
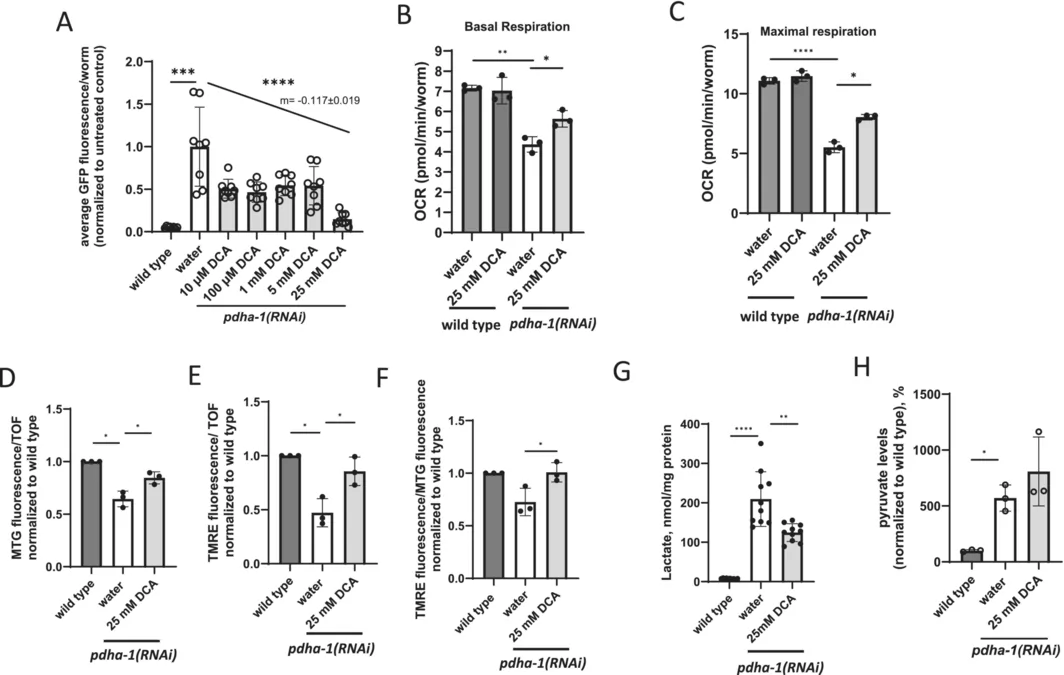
DCA treatment effect on mitochondrial physiology of *pdha‐1(RNAi)* knockdown worms. (A) Untreated *pdha‐1(RNAi)* knockdown worms (white bars) show a significant increase in mitochondrial stress relative to the empty vector control (*hsp‐6p::GFP; myo‐2p::MCherry*, dark gray bar, ***p < 0.001). Treatment with a 5‐log range of DCA concentrations protected animals from UPR^mt^ stress induction (light gray bars, one‐way ANOVA *p* < 0.0001, post hoc linear trend indicated). Data represent mean ± SD of eight replicates with ~50 animals per replicate and condition. (B, C) Basal (B) and maximal (C) mitochondrial respiratory capacity measured by Seahorse assay were significantly reduced in buffer‐treated *pdha‐1(RNAi)* worms relative to wild type (N2 Bristol). Treatment with 25 mM DCA significantly increased mitochondrial respiration rates in *pdha‐1(RNAi)* knockdown animals, while no significant effect was observed in wild‐type controls. Data are presented as mean ± SD from three biological replicate experiments. (D) Mitochondrial content, measured as relative MTG fluorescence normalized to time of flight, was significantly decreased in untreated *pdha‐1(RNAi)* knockdown worms compared to wild‐ type control (N2, Bristol) and significantly increased following DCA treatment. Data represent mean ± SD of three biological replicate experiments. (E) Mitochondrial membrane potential, measured as relative TMRE fluorescence normalized to time of flight, was reduced in untreated *pdha‐1(RNAi)* knockdown worms compared to wild‐type (N2 Bristol) and was restored upon treatment with 25 mM DCA. Data represent mean ± SD of three biological replicates per condition. (F) Relative membrane potential, defined as TMRE fluorescence divided by MTG fluorescence, was decreased in *pdha‐1(RNAi)* knockdown worms relative to wild‐type (N2 Bristol) and increased after treatment with 25 mM DCA. Data convey mean ± SD of three biological replicate experiments per condition. (G) Tissue lactate levels were significantly increased (43%) in the untreated *pdha‐1(RNAi)* knockdown worms relative to wild‐type (N2 Bristol) and significantly decreased after treatment with 25 mM DCA (*p* = 0.0037). Data represent the mean ± SD of 10 biological replicates per condition, with 1000 animals each. (H) Tissue pyruvate levels were significantly increased in *pdha‐1(RNAi)* knockdown worms compared to wild type (N2 Bristol), both with and without treatment with 25 mM DCA. Bars represent the mean ± SD of three biological replicates per condition of 1000 animals each. *p < 0.05, **p < 0.01, and ****p < 0.0001.

To further assess the impact of DCA on mitochondrial physiology, Seahorse‐based respiration assays were performed on *pdha‐1(RNAi)* and wild‐type worms treated from the embryo stage with either water or 25 mM DCA. Consistent restoration of both basal (Figure 2B) and maximal (Figure 2C) respiration was seen in DCA‐treated *pdha‐1(RNAi)* worms across three biological replicate experiments. No significant changes in mitochondrial respiration were detected after treatment of wild‐type worms with 25 mM DCA (Figure 2B,C). Individual OCR traces for all conditions are provided in Figure S4D. Additionally, the effect of DCA treatment was studied on mitochondrial content and relative mitochondrial membrane potential in the *pdha‐1(RNAi)* worms using BioSorter flow cytometry. Specifically, animals treated with either water (buffer control) or 25 mM DCA were co‐stained with MitoTracker Green (MTG) and tetramethylrhodamine ethyl ester (TMRE) to quantify mitochondrial content and membrane potential, respectively. Knockdown of PDHA‐1 led to a significant mean reduction in both mitochondrial content (36%, Figure 2D) and mitochondrial membrane potential (53%, Figure 2E) as compared to wild‐type worms. Importantly, treatment with 25 mM DCA significantly restored both mitochondrial mean parameters to near wild‐type levels (Figure 2B,C and Figure S5). Similarly, the relative mitochondrial membrane potential normalized for mitochondrial content, calculated based on the TMRE/MTG fluorescence ratio, showed a trend toward reduction in *pdha‐1(RNAi)* worms, which significantly increased following DCA treatment (Figure 2F). Validation of these alterations in mitochondrial physiology was performed by an independent method involving quantification of MTG and TMRE relative fluorescence in these animals using high‐content microscopy (CellInsight CX5). This imaging‐based analysis revealed the same overall trends as were observed by flow cytometry, further supporting the conclusion that both mitochondrial content and mitochondrial membrane potential are reduced in *pdha‐1(RNAi)* worms and restored by DCA treatment (Figure S6B–D). In addition, we assessed mitochondrial morphology by confocal microscopy. Imaging was performed in animals expressing *myo‐3p::GFP(mit)*, and three parameters of mitochondrial network morphology were analyzed: roundness, area of individual mitochondrial profiles, and average branch length. Representative confocal images at 63× magnification showed that wild‐type mitochondria formed a predominantly tubular, elongated network, whereas *pdha‐1(RNAi)* animals displayed a more punctate, smaller, fragmented, and disorganized mitochondrial network, with shorter and less continuous structures (Figure S7A). Higher‐magnification images further highlighted these differences, showing shorter and less continuous mitochondrial structures in the *pdha‐1(RNAi)* worms (Figure S7A). Consistent with these observations, quantitative analyses showed that mitochondrial roundness was significantly increased in the *pdha‐1 (RNAi)* animals relative to wild type, indicating a more rounded, less elongated mitochondrial morphology (Figure S7A,B). In addition, mitochondrial profile area and average mitochondrial branch length were significantly decreased (Figure S7C,D) in the *pdha‐1 (RNAi)* animals compared to wild type, suggesting a network composed of small, rounded, poorly branched mitochondrial structures. DCA‐treated animals showed an increase in elongated, tubular mitochondria with fewer punctate structures (Figure S7A). Consistent with this observation, quantitative analyses showed that DCA treatment significantly reduced mitochondrial roundness in *pdha‐1(RNAi)* animals compared to water‐treated controls (Figure S7B) and significantly increased average branch length (Figure S7D), suggesting a partial restoration of mitochondrial network connectivity, while mitochondrial profile area remained unchanged (Figure S7C).

PDHc catalyzes the conversion of pyruvate to acetyl‐coenzyme A, which enters the tricarboxylic acid (TCA) cycle. Reduced PDHc activity leads to pyruvate accumulation, which is subsequently reduced to lactate through the lactate dehydrogenase (LDH) equilibrium reaction. Accordingly, PDCD patients commonly exhibit elevated plasma lactate and pyruvate levels. We therefore measured these metabolites in tissues from *pdha‐1(RNAi)* knockdown worms to determine whether similar metabolic alterations occur. As anticipated, lactate (Figure 2G) and pyruvate (Figure 2H) metabolite levels were significantly increased in the *pdha‐1(RNAi)* whole‐worm populations. Interestingly, 25 mM DCA treatment throughout worm development caused a significant decrease in tissue homogenate lactate in *pdha‐1(RNAi)* worms (*p* = 0.0037, Figure 2G), but did not significantly alter the *pdha‐1(RNAi)* tissue homogenate level of pyruvate (Figure 2H). While the pyruvate: lactate ratio was increased in *pdha‐1(RNAi)* worms (*p* < 0.05), 25 mM DCA treatment did not significantly rescue this biochemical ratio (Figure S6E), as is consistent with no anticipated effect of DCA on the NAD^+^/NADH cytosolic redox ratio.

### 3.2 DCA Treatment Significantly Rescued Growth, Neuromuscular Activity, and Survival in *dld‐1(RNAi)* Worms

DLD‐1 encodes the E3 subunit of several keto acid dehydrogenases, including PDHc that has been previously modeled by RNAi knockdown in *C. elegans* [11, 45], with varying phenotypic severity directly correlating with the degree of RNAi‐induced DLD‐1 knockdown. To model the therapeutic efficacy of DCA on a wide range of severity of PDCD in *C. elegans* , a graded dilution of *dld‐1(RNAi)* bacteria with bacteria harboring an L4440 control vector was used at ratios of 1:100 and 1:20. The varying degree of DLD‐1 knockdown by RNAi dilution was confirmed by the correlated reduction of DLD‐1 protein expression in whole‐worm lysates by western immunoblotting (Figure 3A,B and Figure S8B). As described above, treatment with DCA was performed from the embryo phase, and worms were monitored for potential toxicity at L4 + 1 and L4 + 5 days of adulthood. No toxic effects were observed (Figures S9 and S10). Similar to previous reports [11, 45], lifespan analyses showed that *dld‐1(RNAi)* knockdown worms had increased lifespan, as measured in N2 Bristol by the WormScan method in the presence of FUDR (Figure 3C) and a standard manual lifespan assay without FUDR (Figure S11A). Additionally, the *dld‐1(1:20 RNAi)* knockdown strain showed a significant decrease in lifespan relative to the wild‐type control worms as measured by both WormScan (Figure 3D, *p* = 0.018) and manual lifespan assay (Figure S11A, *p* = 0.002). In contrast, the *dld‐1(1:100 RNAi)* worms showed significantly decreased lifespan when assessed using the WormScan method (Figure S11B, *p* < 0.001), whereas no significant change in lifespan relative to wild‐type control was observed using the standard manual lifespan assay (Figure S11C). Interestingly, 25 mM DCA treatment trended to increase survival in *dld‐1(RNAi)* (Figure 3C and Figure S11D) and significantly extended survival in the *dld‐1(1:20 RNAi)* worms (Figure 3D and Figure S11A), but did not alter lifespans of the *dld‐1(1:100 RNAi)* strain (Figure S11B,C). Thus, a survival benefit from DCA treatment was seen in animals with a more severe inhibition of DLD expression, consistent with our previou ***s*** report using laboratory‐grade DCA [11].

**FIGURE 3:**
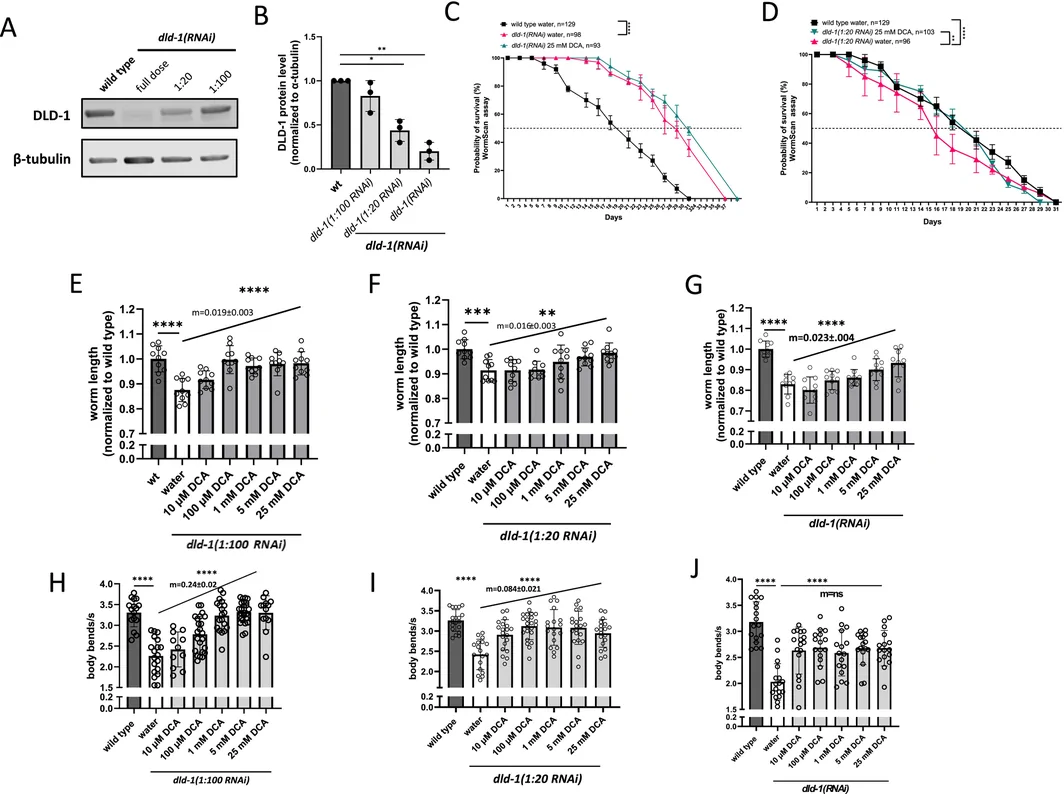
DCA treatment effect on overall animal health of *dld‐1(RNAi)* graded severity knockdown worms. (A) Western blot analysis of DLD‐1 protein levels in *C. elegans* fed with *dld‐1(RNAi)* bacteria (undiluted, 1:20 and 1:100 dilution with L4440) and L4440 (wild‐type, N2 Bristol). A representative blot of *n* = 3 biological replicates is presented. (B) Quantification analysis of DLD‐1 protein band densities in DLD‐1 knockdown worms shows that feeding worms with increasing concentrations of *dld‐1(RNAi)* bacteria leads to a consistent decrease in DLD‐1 protein levels in total worm lysates relative to wild type (N2 Bristol). The levels of DLD‐1 relative to β‐tubulin were normalized to the wild‐type control. Data represent the mean ± SD of three biological replicates. (C) Lifespan analysis of the *dld‐1(RNAi)* worms in the presence of FUDR shows a significant increase (pink curve, *p* < 0.0001) relative to the empty vector control (N2 Bristol, black curve). Treatment with 25 mM DCA caused a trend toward increased lifespan (teal curve). Three biological replicates are presented. (D) Lifespan analysis of the *dld‐1 (1:20) RNAi* worms (pink curve) in the presence of FUDR shows a significant decrease in survival relative to the wild‐type worms (N2 Bristol, black curve, *p* = 0.018), whereas treatment with 25 mM DCA caused a significant increase in lifespan (teal curve, *p* < 0.0001). Three biological replicates are presented. (E–G) Worm growth analysis of *dld‐1(RNAi)* knockdown worms showed a significant decrease in worm length for all *dld‐1(RNAi)* bacteria dilutions tested (white bars) relative to wild type (N2 Bristol). Treatment with DCA significantly rescued growth in all *dld‐1(RNAi)* knockdown worms. All three RNAi dilutions showed a significant dose‐dependent log linear response by one‐way ANOVA. Bars represent the mean ± SD of 10 individual animals, the slope of the linear regression ± Std Er is shown. Post hoc multiple comparisons showed for *dld‐1(1:100 RNAi)* that every DCA dose except 10 μM demonstrated rescue *p* < 0.0001 (E). For *dld‐1(1:20 RNAi)*, the 5 and 25 mM DCA doses demonstrated significant rescue, *p* = 0.042 and *p* = 0.0048, respectively (F) and *dld‐1(RNAi)* the 5 and 25 mM DCA doses demonstrated significant rescue, *p* = 0.019 and *p* = 0.0003, respectively (G). (H–J) *dld‐1(RNAi)* knockdown worms had significantly decreased worm swimming (neuromuscular) activity (white bars) relative to wild‐type (N2 Bristol, dark gray bars), while treatment with DCA significantly improved this phenotype (light gray bars) at all doses tested. *dld‐1*(*1:100 RNAi*) and *dld‐1*(*1:20 RNAi*) knockdown worms showed a significant dose‐dependent linear response, with a lower slope for the 1:20 RNAi dilution. With the undiluted RNAi dose, significant rescue was observed but no dose dependence was detected. Bars represent the mean value ± SD of 20 individual animals. *p < 0.05, **p < 0.01, ***p < 0.001, and ****p < 0.0001.

*dld‐1(RNAi)* knockdown caused a consistent linear growth defect in all three *dld‐1* knockdown concentrations tested (Figure 3E–G). DCA treatment demonstrated significant dose‐dependent rescue of this phenotype by one‐way ANOVA analysis of *dld‐1(1:100 RNAi)*, *dld‐1(1:20 RNAi)*, and *dld‐1(RNAi)*, *p* < 0.0001, *p* < 0.01, and *p* < 0.0001, respectively (Figure 3E–G). A two‐way ANOVA analysis of these data indicated a highly significant (*p* < 0.0001) effect on worm length based on both DCA dose and RNAi dose. The impact of *dld‐1 (RNAi)* knockdown on worm neuromuscular activity was monitored by thrashing analysis in liquid media, where their body bends per second were quantified. Significantly decreased (*p* < 0.0001) thrashing activity by ~35% was observed in all *dld‐1(RNAi)* knockdown strains relative to wild‐type worms (Figure 3H–J). DCA treatment significantly improved thrashing activity in all three *dld‐1(RNAi)* knockdown strains. One‐way ANOVA analysis of DCA treatment with post hoc analysis of a log‐linear regression trend showed that the dependence on DCA concentration directly correlated with the degree of dilution, where DCA treatment had a greater beneficial effect on rescuing worm neuromuscular activity at the mildest (1:100 dilution) than moderate (1:20 dilution) degree of DLD‐1 inhibition, and no significant dose dependence was seen at the most severe degree of full‐dose *dld‐1(RNAi)*. Similar to the worm length analysis presented above, a two‐way ANOVA of the neuromuscular activity data revealed a strong dependence (*p* < 0.0001) on both RNAi concentration and DCA treatment concentration. In addition, we used an automated assay to assess worm light‐stimulated activity at days 2, 5, and 10 of adulthood, as described above. Untreated *dld‐1(full‐dose RNAi)* worms exhibited an abnormally increased activity compared to wild‐type controls, while treatment with 100 μM or 1 mM DCA reduced locomotor activity toward wild‐type levels (Figure S11E).

### 3.3 DCA Treatment Effect on Mitochondrial Stress Induction Correlated With the Severity of the Disease Phenotype in the Graded RNAi Dilution of *dld‐1(RNAi)* Knockdown Worms

Similarly, as described for *pdha‐1(RNAi)*, mitochondrial stress induction was studied in *dld‐1(RNAi)* knockdown strains in a background of N2 Bristol worms stably carrying two transgenic reporters: *hsp‐6p::gfp* (green) and *myo‐2p::mCherry* (red) (Figure S11A). These double‐transgenic worms were fed three different graded RNAi dilutions, *dld‐1(1:100 RNAi)*, *dld‐1(1:20 RNAi)*, and *dld‐1(RNAi)* bacteria from the embryo phase, with UPR^mt^ stress induction analyzed at L4 + 1 day (first day of adulthood) and normalized to the number of worms detected by mCherry fluorescence. As expected, we observed a dramatic increase in UPR^mt^ stress at all three dilutions of *dld‐1(RNAi)* bacteria tested (Figure 4A–C; *p* < 0.0001). The greatest degree of *hsp‐6p*‐dependent induction was seen in full‐dose RNAi knockdown, with proportionate decreased induction in moderate (1:20) and mild (1:100) RNAi induction (Figure S12B). One‐way ANOVA analysis of the DCA effect with post hoc analysis for a log linear trend revealed that DCA did not alter the UPR^mt^ stress induction level with full dose *dld‐1(RNAi)* knockdown (Figure 4C), but a significant dose–response rescue was observed for the moderate *dld‐1(1:20 RNAi)*, *p* < 0.001, and mildest *dld‐1(1:100 RNAi)*, *p* < 0.0001, knockdown strains (Figure 4B and Figure 4A, respectively), with the DCA dose response being greater for *dld‐1(1:100 RNAi)*. Representative images showing the effect of DCA treatment on mitochondrial unfolded protein response stress are presented in Figure S12A. As described above, mitochondrial respiration was evaluated in *dld‐1(full‐dose RNAi)* animals treated with either water or 25 mM DCA. The *dld‐1(RNAi)* knockdown worms displayed a significant reduction in both basal and maximal respiration relative to the wild‐type control (Figure 4D,E). Treatment with 25 mM DCA significantly improved both basal and maximal respiratory capacity, suggesting a partial restoration of mitochondrial function (Figure 4D,E). Full mitochondrial respiration plots for each condition are shown in Figure S12D. Similar to the confocal analyses of the *pdha‐1(RNAi)* worms, the *dld‐1(RNAi)* knockdown animals showed disruption of the mitochondrial network (Figure S7A). Mitochondrial roundness was significantly increased relative to wild type (Figure S7B), while both mitochondrial profile area and average branch length were significantly decreased (Figure S7C,D), showing that knockdown of *dld‐1* promotes fragmentation and rounding of the mitochondrial network. Treatment with 25 mM DCA significantly decreased mitochondrial roundness and significantly increased average branch length in *dld‐1(RNAi)* animals relative to water‐treated controls, while mitochondrial profile area remained unchanged. Together, these results indicate that DCA treatment partially restores mitochondrial network morphology in the *dld‐1(RNAi)* animals, improving network shape and connectivity with no effect on mitochondrial profile area. We did not observe any significant difference between untreated *dld‐1(RNAi)* and *pdha‐1(RNAi)* animals in mitochondrial roundness (*p* = 0.72), profile area (*p* = 0.13), or average branch length (*p* = 0.76). However, treatment with 25 mM DCA caused a greater increase in *dld‐1(RNAi)* than in *pdha‐1(RNAi)* animals (2.1‐fold vs. 1.6‐fold, respectively).

**FIGURE 4:**
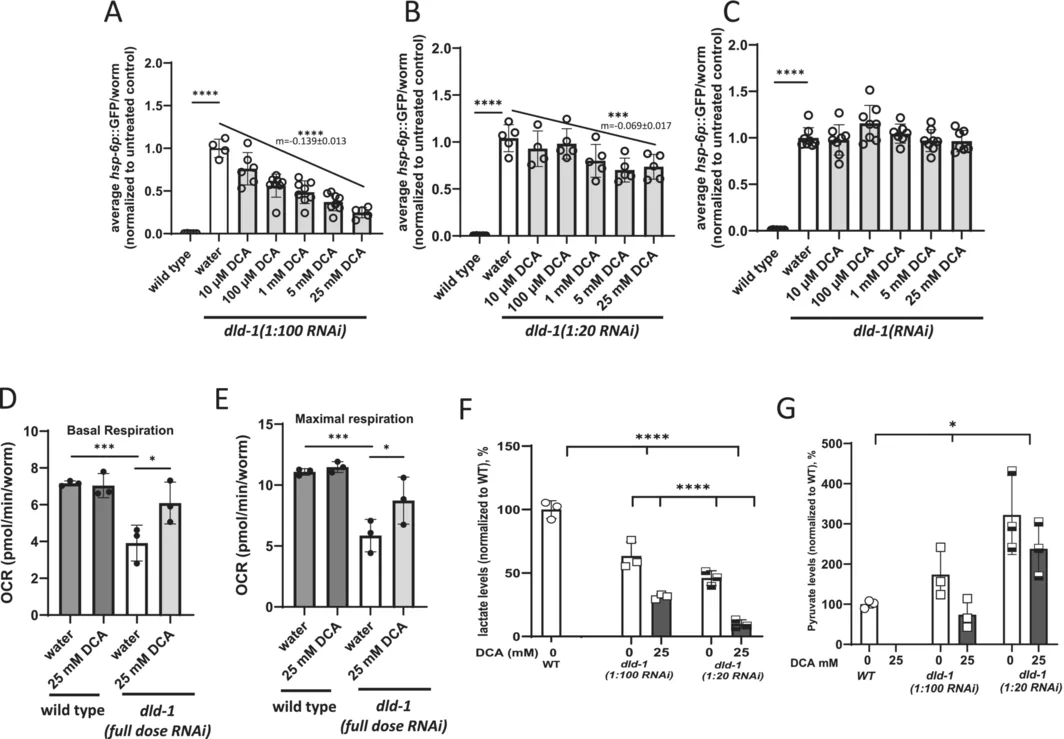
DCA treatment effect on mitochondrial physiology of *dld‐1(RNAi)* graded severity knockdown worms. (A–C) All *dld‐1(RNAi)* knockdown worms (white bars) showed significantly greater induction of the mitochondrial stress response compared to wild‐type control (*hsp‐6p::GFP; myo‐2p::MCherry*, dark gray bars; ****p < 0.0001). Treatment with DCA significantly reduced mitochondrial stress for *dld‐1*(*1:100 RNAi*) and *dld‐1*(*1:20 RNAi*) by one‐way ANOVA, with the log linear regression slope increased from −0.139 to −0.069. With the undiluted *dld‐1*(*RNAi*) no significant effect on mitochondrial stress was observed. Bars represent the mean ± SD of eight replicates with ~50 animals each. (D, E) Oxygen consumption rate (OCR) measured by Seahorse assay reveals reduced basal (D) and maximal (E) respiration in untreated *dld‐1*(full‐dose RNAi) worms relative to wild‐type (N2 Bristol), whereas treatment with 25 mM DCA significantly increased respiration rates. No effect was observed following treatment of wild‐type worms with 25 mM DCA. Data are presented as mean ± SD from three biological replicate experiments. (F) Tissue lactate levels were significantly decreased in *dld‐1(1:20 RNAi)* and *dld‐1(1:100 RNAi)* knockdown worms (white bars) compared to the empty vector control (N2 Bristol, dark gray bars). Further reduction in lactate levels occurred after treatment with 25 mM DCA. Data represent mean ± SD of three biological replicates of 1000 animals each. (G) Tissue pyruvate levels in the *dld‐1* knockdown worms (white bars) trended toward increased in *dld‐1(1:20 RNAi)* by 322% (*p* = 0.058) and in *dld‐1(1:100 RNAi)* by 174% (*p* = 0.063) relative to wild‐type levels (N2 Bristol, dark gray bars). Treatment with 25 mM DCA significantly decreased pyruvate tissue levels (black bars). *p < 0.05, ***p < 0.001, and ****p < 0.0001.

Surprisingly, lactate levels were significantly greater in the mildest (1:100) than moderate (1:20) RNAi dilution worms. 25 mM DCA significantly reduced lactate levels in 1:100 and 1:20 dilution strains of *dld‐1(RNAi)* knockdown (Figure 4F). Pyruvate levels showed an opposite trend, with greater pyruvate levels relative to wild‐type worms evident in a stepwise decreasing fashion in moderate *dld‐1(1:20 RNAi)* than in mild *dld‐1(1:100 RNAi)*, and lowest in full‐dose *dld‐1(RNAi)* (Figure 4G). Similarly, the pyruvate‐to‐lactate ratio was only increased in *dld‐1(1:20 RNAi)* worms but not in the *dld‐1(1:100 RNAi)* worms (Figure S12E). 25 mM DCA showed nonsignificant trends toward reduced pyruvate levels in the *dld‐1(1:100 RNAi)* strain (*p* = 0.085) (Figure 4G) and increased pyruvate: lactate ratio in the moderate *dld‐1(1:20 RNAi)* worms (Figure S11E). As full‐dose *dld‐1(RNAi)* did not have improved mitochondrial stress with DCA treatment, lactate and pyruvate levels were not measured in these conditions.

## 4 Discussion

PDCD is an inherited mitochondrial disorder involving lactic acidosis, neurodevelopmental disability, and high mortality, with treatments largely limited to symptomatic management [46] and clinical trial investigation of DCA [22]. To objectively evaluate the preclinical efficacy of DCA treatment in PDCD, we created and characterized *C. elegans* models of two different genetic causes of PDCD by feeding RNAi to knock down expression of *PDHA‐1* and *DLD‐*1 homologs. Further, *DLD‐1* deficiency was modeled with three different severity grades by titrating the concentration of *dld‐1(RNAi)* bacteria: full dose (severe), 1:20 (moderate), and 1:100 (mild) dilutions. These worm models recapitulate key phenotypic features observed in PDHc patients. Impaired growth, reduced neuromuscular activity, induction of mitochondrial stress, and reduced OCRs were common to both *pdha‐1(RNAi)* and *dld‐1(RNAi)* models, while other phenotypes, such as tissue lactate levels and survival, were dependent on the model or level of knockdown. These differences likely reflect the difference in biology of the two genes rather than differences in experimental approach. PDHA‐1 is the E1 alpha subunit, whereas DLD‐1 is the E3 subunit shared by the pyruvate dehydrogenase, α‐ketoglutarate dehydrogenase, 2‐oxoadipate dehydrogenase, and branched‐chain α‐ketoacid dehydrogenase complexes, as well as the glycine cleavage system within the mitochondrial matrix. Knockdown of *dld‐1* therefore may produce broader metabolic defects than *pdha‐1*.

Extensive phenotypic analyses of both RNAi knockdown models' impact on lifespan, health span, intermediary metabolism, and diverse readouts of mitochondrial physiology were performed at baseline relative to wild‐type worms, and following treatment with DCA across a 5‐point log scale (10 μM to 25 mM) in growth, neuromuscular function, and mitochondrial stress induction studies, and high‐dose (25 mM) DCA in tissue metabolite and lifespan studies. Across all studies, DCA showed no toxic effects at the level of survival, health span (growth, neuromuscular activity), or metabolism (mitochondrial UPR^mt^ stress induction, mitochondrial amount or membrane potential, mitochondrial respiratory capacity, or lactate and pyruvate levels). Indeed, DCA treatment led to statistically significant therapeutic benefit on multiple phenotypic and metabolic outcome measures in both PDHc knockdown *C. elegans* models, including mitochondrial content, membrane potential, respiratory capacity (OCR), stress induction, and morphology, with multiple DCA dose–response effects seen.

Decreasing *pdha‐1* expression in *C. elegans* significantly reduced survival, significantly decreased worm linear growth and neuromuscular activity, and significantly increased mitochondrial stress induction as well as tissue levels of lactate and pyruvate. Additionally, the *pdha‐1* knockdown worms showed decreased respiration rates, mitochondrial content, and membrane potential. DCA treatment showed a statistically significant benefit on animal survival, overall animal health at the level of neuromuscular function (thrashing), and linear growth. In addition, improvements in overall metabolic and mitochondrial physiology upon DCA treatment were evident in the *pdha‐1(RNAi)* strain, including reduced lactate, reduced mitochondrial stress induction, increased mitochondrial membrane potential and mitochondrial content, and enhanced basal and maximal mitochondrial respiratory capacity. Interestingly, while the DCA effect leveled off in some experimental outcomes, no specific dose threshold was universally beneficial in all conditions studied. Survival analyses were performed following treatment with the highest tested DCA dose (25 mM), since this concentration consistently produced beneficial effects across all other assays, including worm length, neuromuscular activity, and notably, reduced UPR^mt^ stress levels to those observed in wild‐type worms. Previously, our research group had studied the intermediary metabolism effects of PDHc RNAi knockdown using stable isotope and mass spectrometry studies [23], further adding to the body of informative data confirming the relevance of this *C. elegans* *pdha‐1(RNAi)* knockdown model to study PDHA1‐based human disease. Treatment with 25 mM DCA in graded models of *dld‐1 RNAi* silencing significantly prolonged animal survival only in the more severe knockdown conditions, namely *dld‐1(RNAi) and dld‐1(1:20 RNAi)*, similarly to previously reported [11, 45], with no effect seen in the *dld‐1(1:100 RNAi)* dilution strain. At baseline, lifespan across the three *dld‐1(RNAi)* strains did not correlate with knockdown severity. The full‐dose *dld‐1(RNAi)* strain had an extended lifespan, while the moderate and mild knockdown strains showed reduced lifespan in the presence of FUDR. This finding is consistent with previous observations from our group and others [11, 47] showing that, under certain conditions, mitochondrial stress can promote longevity in *C. elegans* . Direct comparison of the lifespan results obtained in the present study with those reported in our previous study on PDCD models [11] is presented in Table S1. Interestingly, our prior work has found that in multiple mitochondrial disease models, including *dld‐1(RNAi)* worms, therapies started during development induce a greater degree of outcome rescue than those started in adult‐stage animals. *dld‐1(RNAi)* knockdown significantly reduced worm linear growth and neuromuscular activity in all three knockdown dilutions tested. The reduced linear growth observed in the *dld‐1(RNAi)* strains was not caused by delayed development. All three *dld‐1(RNAi)* strains reached the L4 stage at a time comparable to wild‐type animals, consistent with our previous observations [11]. Therefore, the reduced body size observed at L4 + 1 day suggests impaired organismal growth rather than a delay in development. In addition, we observed an increased induction of mitochondrial stress in the *dld‐1(RNAi)* animals that inversely correlated with the level of residual DLD‐1 protein. However, *dld‐1(1:20 RNAi)* and *dld‐1(1:100 RNAi)* strains showed a trend toward decreased tissue lactate levels and increased tissue pyruvate levels. Importantly, lactate levels were measured in whole‐worm tissue homogenates and therefore reflect the combined intracellular and extracellular levels. In our previous work using a *C. elegans* model of FBXL4 deficiency, we observed a similar decrease in whole‐worm tissue lactate levels, despite increased extracellular lactate in FBXL4‐deficient human fibroblasts. Therefore, the reduced whole‐worm lactate levels observed in the *dld‐1(1:20 RNAi)* and *dld‐1(1:100 RNAi)* strains do not necessarily indicate reduced extracellular lactate production but may reflect altered tissue metabolism wherein lactate can be used as a metabolic fuel. Together with the observed increases in tissue pyruvate and pyruvate: lactate ratio in the *dld‐1(1:20 RNAi)* strain, these findings suggest abnormal pyruvate/lactate metabolism is induced in response to DLD‐1 deficiency. Analysis of DCA treatment in the three *dld‐1(RNAi)* knockdown strains confirms a differential sensitivity to DCA that correlated with the severity of the observed phenotype. While treatment with DCA rescued animal growth and neuromuscular activity in all *dld‐1(RNAi)* strains, we observed distinct responses of UPR^mt^ stress that correlated with the degree of *dld‐1(RNAi)* knockdown. The most severe full‐dose *dld‐1(RNAi)* knockdown strain showed no improvement with DCA, whereas the less severe *1:20* and *1:100 dld‐1(RNAi)* knockdown strains showed an increasing dose‐dependent response after treatment with DCA at tested concentrations. However, treatment with 25 mM DCA restored both basal and maximal mitochondrial respiratory capacity in the *dld‐1 (RNAi)* worms. Additionally, both *dld‐1(RNAi)* and *pdha‐1(RNAi)* animals displayed altered mitochondrial network morphology relative to wild‐type animals, characterized by smaller, more rounded mitochondrial profiles and reduced average branch length. Although loss of DLD‐1 and PDHA‐1 produces distinct metabolic consequences, their mitochondrial network architecture effects were comparable in both models. Importantly, treatment with 25 mM DCA normalized this network architecture, as manifested by reduced roundness and increased average branch length in both models' mitochondria.

These data add to the preclinical literature in PDHc models of DCA, a structural analog of pyruvate that has been previously shown to rescue disease phenotypes in some PDHc models [11] while having no effect in others [12, 15, 21]. The reason for this differential effect was proposed to be the nature of the E1 alpha subunit mutations, with mutations affecting the stability of the E1 alpha subunit being responsive, while mutations that affect the catalytic activity of the protein being unresponsive to DCA treatment [15]. Our prior studies in a very severe zebrafish DLD knockout model that had very low residual PDHc activity and larval mortality had no clear phenotypic benefit from DCA, although a metabolic improvement was seen [12]. We postulate that this may relate to DCA requiring a certain minimum expression of PDHc to have therapeutic benefit, as it activates residual PDHc and, in the setting of severe DLD knockout, would anticipate insufficient substrate present for meaningful activation of residual PDHc activity by DCA. Consistent with this hypothesis, studies in the *C. elegans* *dld‐1* models presented here revealed significant therapeutic benefit of DCA on survival, neuromuscular and metabolic phenotypes primarily in the moderate (1:20) and/or mild (1:100) *dld‐1* dilution strains, rather than the full‐dose *dld‐1(RNAi)*, although linear animal growth and neuromuscular activity were significantly improved in all three RNAi strains studied upon DCA treatment.

Collectively, these preclinical evaluations demonstrate objective therapeutic benefit of DCA in two distinct genetic etiologies of PDCD, namely homologs of *PDHA1* and *DLD*. Additional insight is provided into the selective sensitivity of PDCD models to DCA, showing that beneficial effects in rescuing mitochondrial stress that is significantly induced in the *dld‐1 (RNAi)* models are inversely correlated with disease severity. Importantly, these preclinical data serve as confirmatory evidence to support data from a single completed Phase 3 clinical trial to evaluate the therapeutic utility of DCA in PDCD, with demonstrated beneficial effects on survival, healthspan, tissue lactate, and diverse aspects of mitochondrial physiology. While that clinical trial excluded *DLD* deficiency patients due to the multi‐enzyme deficiency caused by the E3 subunit being a component of multiple mitochondrial keto acid dehydrogenases beyond PDHc, these preclinical data in *dld‐1(RNAi)* knockdown worms suggest that survival, healthspan, as well as diverse metabolic disruptions that occur in mild to moderate degrees of *DLD* deficiency do significantly improve with DCA treatment.

## Author Contributions

M.J.F. conceived of the study, obtained funding, and oversaw study performance. C.R. performed *C. elegans* growth, sequencing, phenotyping, and treatment studies. V.M., C.R., and N.D.M. performed *C. elegans* lifespan studies. C.R. and V.M. performed the western blot analysis. T.O. performed respiration analyses studies. S.H. performed the confocal imaging experiments and assisted with analysis of both Seahorse and confocal microscopy data. M.L. performed confocal microscopy data analyses. K.K. analyzed the automatic worm locomotor activity data. E.N.‐O. performed lactate and pyruvate analyses. R.X. provided statistical review and guidance. C.R. and V.E.A. analyzed the data, performed statistical analyses, and wrote the initial manuscript draft with M.J.F. All authors reviewed and approved the final manuscript version.

## Funding

This study was supported by Saol Therapeutics and the National Institutes of Health (R35‐GM134863).

## Ethics Statement

The authors have nothing to report.

## Consent

The authors have nothing to report.

## Conflicts of Interest

M.J.F. is inventor of US patent 12,011,452 B2 issued Jun 18, 2024, “Compositions and Methods for Treatment of Mitochondrial Respiratory Chain Dysfunction and Other Mitochondrial Disorders”. M.J.F. is engaged with companies involved in mitochondrial disease therapeutic preclinical and/or clinical‐stage development. M.J.F. is co‐founder of Rarefy Therapeutics; an advisory board member with equity interest in RiboNova Inc.; a scientific advisory board member and paid consultant with Khondrion and Larimar Therapeutics; has been a paid consultant for Ajinomoto‐Cambrooke Inc., Astellas (formerly Mitobridge), BPGbio, Casma Therapeutics, Cyclerion Therapeutics, Epirium Bio (formerly Cardero Therapeutics), HealthCap VII Advisor AB, Imel Therapeutics, Mayflower Inc., Primera Therapeutics Inc., Minovia Therapeutics, Mission Therapeutics, NeuroVive Pharmaceutical AB, Precision Biosciences, Reneo Therapeutics, Saol Therapeutics, Sironax, Stealth BioTherapeutics, Vincere Bio, and Zogenix; and/or has been a sponsored research collaborator for Aadi Bioscience, Adjuvia Therapeutics, Astellas, Cyclerion Therapeutics, Epirium Bio, Imel Therapeutics, Khondrion, Merck, Minovia Therapeutics, Mission Therapeutics, NeuroVive Pharmaceutical AB, Precision Biosciences, Pretzel Therapeutics, PTC Therapeutics, Raptor Pharmaceuticals, REATA Inc., Reneo Therapeutics, RiboNova, Saol Therapeutics, Standigm, Stealth BioTherapeutics, and Thiogenesis. M.J.F. also has received royalties from Elsevier and speaker fees from Agios Pharmaceuticals, GenoMind, and educational honoraria from PlatformQ and Chemistry Rx. No other co‐author has a relevant conflicts of interest to declare.

## Supporting information


**Figure S1:** (A) Amino acid alignment between the human PDHA1 and *C. elegans* PDHA‐1 proteins. (B) Uncropped western blot images corresponding to Figure 1A, together with two additional biological replicates used for PDHA‐1 protein quantification in Figure 1B. (C) Lifespan analysis of wild‐type worms (N2 Bristol) treated with 25 mM DCA shows no significant differences compared with untreated controls, measured by the WormScan assay, in the presence of FUDR. (D) Manual lifespan analysis without FUDR showed a significant reduction in lifespan upon *pdha‐1(RNAi)* knockdown relative to the wild‐type (N2 Bristol) control (*p* = 0.0012), whereas treatment with 25 mM DCA restored lifespan in *pdha‐1(RNAi)* knockdown worms toward wild‐type (N2 Bristol) levels (*p* = 0.013), across three biological replicates. (E) Stimulated locomotor activity of wild‐type (N2 Bristol) and *pdha‐1(RNAi)* worms measured using an automated system, under untreated conditions and across a five‐point DCA dose series. Analyses are shown at L4 + 2, L4 + 5, and L4 + 10 days of adulthood. Activity values were normalized to the corresponding wild‐type control on each plate. Data represent the mean ± SD of six technical replicates with ~20 animals each. (F) Log‐linear regression analysis of DCA dose–response on worm length, demonstrating a significant positive dose–response relationship between DCA concentration and normalized worm length.
**Figure S2:** Microscopy images of wild‐type (N2 Bristol) (A) and *pdha‐1(RNAi)* knockdown (B) worms treated with water (buffer control) or a four‐point DCA dose series, imaged at L4 + 1 day. Scale bar: 200 μm.
**Figure S3:** Microscopy images of wild‐type (N2 Bristol) (A) and *pdha‐1(RNAi)* knockdown (B) worms treated with water (buffer control) or a five‐point DCA dose series, imaged at L4 + 5 days. Scale bar: 200 μm.
**Figure S4:** (A) Representative CX5 images for worms fed with empty vector (wild‐type, *hsp‐6p::GFP; myo‐2p::mCherry*) and *pdha‐1*(*RNAi*) bacteria, treated with water or 25 mM DCA. The red fluorescence (mCherry) was used to determine the number of animals per well, and the green fluorescence (GFP) was used to quantify the level of UPR^mt^. (B, C) FCCP titration curve was performed on untreated wild‐type (N2 Bristol) (B) and untreated *pdha‐1(RNAi)* (C) animals to determine the optimal FCCP concentration for Seahorse analyses. (D) Oxygen consumption rate (OCR) of wild‐type (N2 Bristol) and *pdha‐1(RNAi)* knockdown animals treated with water (buffer control) or 25 mM DCA, measured by Seahorse assay following sequential addition of two mitochondrial function modulators: FCCP (25 μM) and sodium azide (50 mM). Data represent the mean ± SD of 15 technical replicates per biological replicate (with ~75 animals/well). (E) Non‐mitochondrial OCR measured for the wild‐type (N2 Bristol), *pdha‐1(RNAi)*, and *dld‐1(RNAi)* worms, treated with water or 25 mM DCA, measured across three biological replicates. Data represent the mean ± SD of 3 replicates with 15 technical replicates each.
**Figure S5:** Representative BioSorter dot plots showing time of flight (TOF) versus green fluorescence (left panels) and red fluorescence (right panels) for wild‐type worms (N2 Bristol) (A), *pdha‐1(RNAi)* worms (B), and *pdha‐1(RNAi)* worms treated with 25 mM DCA (C). Each dot represents an individual animal. All objects detected were included in the analysis; no gating was applied. Panels shown are representative of three independent biological replicate experiments per condition.
**Figure S6:** (A–C) CX5 analysis of wild‐type (*hsp‐6p::GFP; myo‐2p::mCherry*) and *pdha‐1(RNAi)* knockdown worms treated with water or 25 mM DCA and co‐labeled with TMRE and MTG. Average red (B) and green (C) fluorescence of *pdha‐1(RNAi)* animals are shown, normalized to wild‐type levels. Data represent the mean ± SD of four replicates with ~50 animals each. (D) CX5 analysis of the relative mitochondrial membrane potential, calculated as the ratio of TMRE (red channel) fluorescence to MTG (green channel) fluorescence, relative to wild‐type (N2 Bristol). Data represent the mean ± SD of four replicates with ~50 animals each. (E) Pyruvate/Lactate ratio in the *pdha‐1(RNAi)* knockdown worms was increased relative to wild‐type (N2 Bristol), while treatment with DCA did not significantly alter this ratio. *p < 0.05.
**Figure S7:** Quantification of mitochondrial morphology in body‐wall muscle of wild‐type, *dld‐1*, and *pdha‐1* worms at baseline and after treatment with 25 mM DCA. (A) Representative images illustrating mitochondrial morphology in body‐wall muscle. The upper row shows 63× images of mitochondrial networks in wild‐type, *dld‐1(RNAi)*, and *pdha‐1(RNAi)* worms. The corresponding images shown below each condition are higher‐magnification views highlighting the differences in mitochondrial morphology. In wild‐type worms, mitochondria predominantly exhibited a tubular and elongated morphology, whereas both *dld‐1(RNAi)* and *pdha‐1(RNAi)* animals displayed a more punctate and disorganized morphology, characterized by shorter and less continuous mitochondrial structures. The arrowhead indicates a representative tubular/elongated mitochondrial structure in wild type, while the dashed rectangles highlight representative regions showing the altered, punctate/disorganized morphology in the knockdown animals. Treatment with 25 mM DCA resulted in a shift toward a more elongated and tubular mitochondrial morphology in both *dld‐1(RNAi)* and *pdha‐1(RNAi)* animals, as illustrated in the corresponding images below. (B) Both *pdha‐1* and *dld‐1* knockdown strains exhibited increased mitochondrial roundness compared with wild‐type worms. Treatment with 25 mM DCA significantly reduced mitochondrial roundness in both *pdha‐1* and *dld‐1* worms, restoring the values to levels comparable to wild type. (C) Quantification of mitochondrial profile area revealed a modest but significant reduction in *dld‐1* and *pdha‐1* knockdown worms compared with wild type. DCA treatment did not significantly affect mitochondrial profile area in either strain. (D) Quantification of average mitochondrial branch length showed that both *dld‐1* and *pdha‐1* worms have significantly reduced branch length compared with wild type. Treatment with 25 mM DCA significantly increased average mitochondrial branch length in both strains. Data are shown as mean ± SD, each point representing a single mitochondrial profile. Statistical significance was determined using one‐way ANOVA with Šídák's multiple comparisons test; **p < 0.01; ***p < 0.001; ****p < 0.0001; ns, not significant.
**Figure S8:** (A) Amino acid alignment between the human DLDH and *C. elegans* DLD‐1 protein. (B) Uncropped western blot images corresponding to Figure 3A, together with two additional biological replicates used for quantification of the DLD‐1 protein in Figure 3B.
**Figure S9:** Microscopy images of *dld‐1*, *dld‐1 (1:20)*, and *dld‐1 (1:100) RNAi* worms treated with water (buffer control) or a five‐point DCA dose series, imaged at L4 + 1 day. Scale bar: 200 μm.
**Figure S10:** Microscopy images of *dld‐1*, *dld‐1 (1:20)*, and *dld‐1 (1:100) RNAi* worms treated with water (buffer control) or a five‐point DCA dose series, imaged at L4 + 5 days. Scale bar: 200 μm.
**Figure S11:** (A) Manual lifespan analysis without FUDR showed that *dld‐1(1:20 RNAi)* worms had significantly reduced survival compared to wild‐type controls (N2 Bristol, *p* < 0.0001), whereas treatment with 25 mM DCA significantly increased their survival (*p* < 0.0001) across three biological replicates. (B) Lifespan analysis using the WormScan method in the presence of FUDR showed that *dld‐1(1:100 RNAi)* worms had significantly reduced survival compared to wild‐type controls (N2 Bristol, *p* = 0.002), whereas treatment with 25 mM DCA had no effect on survival. Three biological replicates are presented. (C) Lifespan analysis using a standard manual assay without FUDR showed no difference in survival of *dld‐1(1:100 RNAi)* knockdown worms compared to wild‐type controls (N2 Bristol), and treatment with 25 mM DCA did not affect survival. Three biological replicates are presented. (D) Lifespan analysis using a standard manual assay without FUDR at 20°C showed that *dld‐1* knockdown worms had significantly increased survival compared to wild‐type controls (N2 Bristol, *p* < 0.0001), while treatment with 25 mM DCA further significantly extended their survival (*p* = 0.01). Three biological replicates are presented. (E) Light‐stimulated locomotor activity of wild‐type (N2 Bristol, WT) and *dld‐1* worms measured using an automated system under untreated conditions and across a four‐point DCA dose series. Analyses are shown for the L4 + 2, L4 + 5, and L4 + 10 days of adulthood. Activity values were normalized to the corresponding wild‐type control on each plate. Data represent the mean ± SD of six technical replicates with ~20 animals each.
**Figure S12:** (A) Representative CX5 images for worms fed with L4440 (*hsp‐6p::GFP + myo‐2p::mCherry*, wild type) and *dld‐1(RNAi)*, *dld‐1(1:20 RNAi)*, and *dld‐1(1:100 RNAi)* bacteria treated with water or 25 mM DCA. The red fluorescence (mCherry) was used to determine the number of animals per well, and the green fluorescence (GFP) was used to quantify the level of mitochondrial stress induction. (B) Quantification of mitochondrial stress upon feeding worms with increasing concentrations of *dld‐1* RNAi bacteria. Data represent the mean ± SD of 8 replicate experiments with ~50 animals per condition. (C) An FCCP titration curve was performed in untreated *dld‐1(RNAi)* knockdown animals to determine the optimal FCCP concentration for Seahorse analyses. Data represent the mean ± SD of 15 technical replicates per biological replicate, with one animal per condition. (D) OCR of wild‐type (N2 Bristol) and *dld‐1(RNAi)* knockdown animals treated with water (buffer control) or 25 mM DCA, measured by Seahorse assay following sequential addition of mitochondrial function modulators: FCCP (25 μM) and sodium azide (50 mM). Data represent the mean ± SD of 15 technical replicates per biological replicate, with one animal per condition. (E) Pyruvate/Lactate ratio in *dld‐1*(*1:20* RNAi) knockdown worms trended toward increase relative to wild type (N2 Bristol), and treatment with 25 mM DCA trended to further increase this ratio. *dld‐1(1:100 RNAi*) worms showed no difference in pyruvate/lactate levels compared to wild type, and treatment with DCA did not significantly change this ratio. ****p < 0.0001.
**Table S1:** Comparison of lifespan data, measured using the WormScan method, in the presence of FUDR, for *dld‐1(RNAi)* knockdown worms from this study and previous reports.
**Table S2:** Overview of the experimental design and conditions presented in this study.

## Data Availability

The data that support the findings of this study are available in the Supporting Information of this article.
