# Supplementary material for: Dichloroacetate Improves Animal Survival, Growth, Neuromuscular Activity, Mitochondrial Stress and Physiology, and Elevated Lactate in C. elegans pdha‐1 and dld‐1 RNAi Models of Pyruvate Dehydrogenase Complex Deficiency (PDCD)
Source: J Inherit Metab Dis. 2026 Oct 7;49(6):e70257. doi: 10.1002/jimd.70257 (PMC13646854; doi:10.1002/jimd.70257)
Supplement: Supplementary file 1 [file JIMD-49-0-s001.docx]

**SUPPLEMENTAL FILES**

**Dichloroacetate improves animal survival, growth, neuromuscular activity, mitochondrial stress and physiology, and elevated lactate in *C. elegans pdha-1* and *dld-1* RNAi models of pyruvate dehydrogenase complex deficiency (PDCD)**

Cristina Remes^1^, Neal D. Mathew^1^, Victoria Miranda^1^, Suraiya Haroon^1,2^, Tyler O’Hara^1^, Vernon E. Anderson^1^, Manuela Lavorato^1,2^, Kelsey Keith^3^, Rui Xiao^4^, Eiko Nakamaru-Ogiso^1,2^, Marni J. Falk^1,2^*

^1^ Mitochondrial Medicine Frontier Program, Division of Genetic and Genomic Medicine, Department of Pediatrics, The Children’s Hospital of Philadelphia (CHOP), Philadelphia, Pennsylvania, USA.

^2^Department of Pediatrics, Perelman School of Medicine, University of Pennsylvania, Philadelphia, Pennsylvania, USA

^3^Department of Biomedical and Health Informatics, The Children’s Hospital of Philadelphia, Philadelphia, PA

^4^Department of Biostatistics, Perelman School of Medicine, University of Pennsylvania, Philadelphia,

*Corresponding author

**SUPPLEMENTAL FILE LEGENDS**

**Supplementary Fig. 1. (A)** Amino acid alignment between the human PDHA1and *C. elegans* PDHA-1 proteins. **(B)** Uncropped western blot images corresponding to Fig. 1A, together with two additional biological replicates used for PDHA-1 protein quantification in Fig. 1B. **(C)** Lifespan analysis of wild-type worms (N2 Bristol) treated with a 25 mM DCA shows no significant differences compared with untreated controls, measured by the WormScan assay, in presence of FUDR. **(D)** Manual lifespan analysis without FUDR showed significant reduction in lifespan upon *pdha-1(RNAi)* knockdown relative to the wild-type (N2 Bristol) control (p=0.0012), whereas treatment with 25 mM DCA restored lifespan in *pdha-1(RNAi)* knockdown worms toward wild-type (N2 Bristol) levels (p=0.013), across three biological replicates. **(E)** Stimulated locomotor activity of wild-type (N2 Bristol) and *pdha-1(RNAi)* worms measured using an automated system, under untreated conditions and across a four-point DCA dose series. Analyses are shown for stages L4+2 days, L4+5 days, and L4+10 days of adulthood. Activity values were normalized to the corresponding wild-type control on each plate. Data represent the mean ± SD of 6 biological replicates with ~20 animals each. **(F**) Log-linear regression analysis of DCA dose–response on worm length, demonstrating a significant positive dose–response relationship between DCA concentration and normalized worm length.

**Supplementary Fig. 2.** Microscopy images of wild-type (N2 Bristol) (A) and *pdha-1(RNAi)* knockdown (B) worms treated with water (buffer control) or a four-point DCA dose series, imaged at stage L4+1 day. Scale bar: 200 µm

**Supplementary Fig. 3.** Microscopy images of wild-type (N2 Bristol) (A) and *pdha-1(RNAi)* knockdown (B) worms treated with water (buffer control) or a four-point DCA dose series, imaged at stage L4+5 day. Scale bar: 200 µm

**Supplementary Fig. 4. (A)** Representative CX5 images for worms fed with empty vector (wild-type, *hsp-6p::GFP; myo-2p::mCherry*) and *pdha-1*(*RNAi*) bacteria, treated with water or 25 mM DCA. The red fluorescence (mCherry) was used to determine the number of animals per well, and the green fluorescence (GFP) was used to quantify the level of UPR^mt^. **(B-C)** FCCP titration curve was performed untreated wild-type (N2 Bristol) (B) and untreated *pdha-1(RNAi)* (C) animals to determine the optimal FCCP concentration for Seahorse analyses. **(D)** Oxygen consumption rate (OCR) of wild-type (N2 Bristol) and *pdha-1(RNAi)* knockdown animals treated with water (buffer control) or 25 mM DCA, measured by Seahorse assay following sequential addition of two mitochondrial function modulators: FCCP (25 µM) and sodium azide (50 mM). Data represent the mean ± SD of 15 technical replicates per biological replicate (with ~75 animals/well). **(E)** Non-mitochondrial OCR measured for the wild-type (N2 Bristol), *pdha-1(RNAi)*, and *dld-1(RNAi)* worms, treated with water or 25 mM DCA, measured across 3 biological replicates. Data represent the mean ± SD of 3 replicates with 15 technical replicates each.

**Supplementary Fig. 5.** Representative Biosorter dot plots showing time of flight (TOF) versus green fluorescence (left panels) and red fluorescence (right panels) for wild-type worms (N2 Bristol) **(A)**, *pdha-1(RNAi)* worms **(B)**, and *pdha-1(RNAi)* worms treated with 25 mM DCA **(C)**. Each dot represents an individual animal. All objects detected were included in the analysis; no gating was applied. Panels shown are representative of three independent biological replicate experiments per condition.

**Supplementary Fig. 6. (A-C)** CX5 analysis of wild-type (*hsp-6p::GFP; myo-2p::mCherry*) and *pdha-1(RNAi)* knockdown worms treated with water or 25 mM DCA and co-labeled with TMRE and MTG. Average red **(B)** and green **(C)** fluorescence of *pdha-1(RNAi)* animals are shown, normalized to wild-type levels. Data represent the mean ± SD of 4 replicates with ~50 animals each. **(D)** CX5 analysis of the relative mitochondrial membrane potential, calculated as the ratio of TMRE (red channel) fluorescence to MTG (green channel) fluorescence, relative to wild-type (N2 Bristol). Data represent the mean ± SD of 4 replicates with ~50 animals each. **(E)** Pyruvate/Lactate ratio in the *pdha-1(RNAi)* knockdown worms was increased relative to wild-type (N2 Bristol), while treatment with DCA did not significantly alter this ratio. *: p <0.05

**Supplementary Fig. 7.** Quantification of mitochondrial morphology in body-wall muscle of wild type, *dld-1* and *pdha-1* worms at baseline and after treatment with 25 mM DCA. **(A)** Representative images illustrating mitochondrial morphology in body-wall muscle. The upper row shows 63× images of mitochondrial networks in wild type, *dld-1(RNAi)*, and *pdha-1(RNAi)* worms. The corresponding images shown below each condition are higher-magnification views highlighting the differences in mitochondrial morphology. In wild type worms, mitochondria predominantly exhibited a tubular and elongated morphology, whereas both *dld-1(RNAi)* and *pdha-1(RNAi)* animals displayed a more punctate and disorganized morphology, characterized by shorter and less continuous mitochondrial structures. The arrowhead indicates a representative tubular/elongated mitochondrial structure in wild type, while the dashed rectangles highlight representative regions showing the altered, punctate/disorganized morphology in the knockdown animals. Treatment with 25 mM DCA resulted in a shift toward a more elongated and tubular mitochondrial morphology in both *dld-1(RNAi)* and *pdha-1(RNAi)* animals, as illustrated in the corresponding images below. **(B)** Both *pdha-1* and *dld-1* knockdown strains exhibited increased mitochondrial roundness compared with wild-type worms. Treatment with 25 mM DCA significantly reduced mitochondrial roundness in both *pdha-1* and *dld-1* worms, restoring the values to levels comparable to wild type. **(C)** Quantification of mitochondrial profile area revealed a modest but significant reduction in *dld-1* and *pdha-1* knockdown worms compared with wild type. DCA treatment did not significantly affect mitochondrial profile area in either o strain. **(D)** Quantification of average mitochondrial branch length showed that both *dld-1* and *pdha-1* worms have significantly reduced branch length compared with wild type. Treatment with 25 mM DCA significantly increased average mitochondrial branch length in both strains. Data are shown as mean ± SD, each point representing a single mitochondrial profile. Statistical significance was determined using One way ANOVA with Šídák's multiple comparisons test; **p<0.01; ***p<0.001; ****p<0.0001; ns, not significant.

**Supplementary Fig. 8. (A)** Amino acid alignment between the human DLDH and *C. elegans* dld-1 protein. **(B)** Uncropped western blot images corresponding to Fig. 3A, together with two additional biological replicates used for quantification of the DLD-1 protein in Fig. 3B.

**Supplementary Fig. 9.** Microscopy images of *dld-1, dld-1 (1:20) and dld-1 (1:100) RNAi* worms treated with water (buffer control) or a four-point DCA dose series, imaged at the stage L4+1 day. Scale bar: 200 µm

**Supplementary Fig. 10.** Microscopy images of *dld-1, dld-1 (1:20) and dld-1 (1:100) RNAi* worms treated with water (buffer control) or a four-point DCA dose series, imaged at stage L4+5 days. Scale bar: 200 µm

**Supplementary Fig. 11. (A)** Manual lifespan analysis without FUDR showed that *dld-1(1:20 RNAi)* worms had significantly reduced survival compared to wild-type controls (N2 Bristol, p<0.0001), whereas treatment with 25 mM DCA significantly increased their survival (p<0.0001) across three biological replicates. **(B)** Lifespan analysis using the WormScan method in presence of FUDR showed that *dld-1(1:100 RNAi)* worms had significantly reduced survival compared to wild-type controls (N2 Bristol, p=0.002), whereas treatment with 25 mM DCA had no effect on survival. Three biological replicates are presented. **(C)** Lifespan analysis using a standard manual assay without FUDR showed no difference in survival of *dld-1(1:100 RNAi)* knockdown worms compared to wild-type controls (N2 Bristol), and treatment with 25 mM DCA did not affect survival. Three biological replicates are presented. **(D)** Lifespan analysis using a standard manual assay without FUDR at 20ºC showed that *dld-1* knockdown worms had significantly increased survival compared to wild-type controls (N2 Bristol, p<0.0001), while treatment with 25 mM DCA further significantly extended their survival (p=0.01). Three biological replicates are presented. **(E)** Light stimulated locomotor activity of wild-type (N2 Bristol, WT) and *dld-1* worms measured using an automated system under untreated conditions and across a four-point DCA dose series. Analyses are shown for the L4+2 days, L4+5 days, and L4+10 days of adulthood. Activity values were normalized to the corresponding wild-type control on each plate. Data represent the mean ± SD of 6 technical replicates with ~20 animals each.

**Supplementary Fig. 12. (A)** Representative CX5 images for worms fed with L4440 (*hsp-6p::GFP + myo-2p::mCherry*, wild type) and *dld-1(RNAi)*, *dld-1(1:20 RNAi)*, and *dld-1(1:100 RNAi)* bacteria treated with water or 25 mM DCA. The red fluorescence (mCherry) was used to determine the number of animals per well, and the green fluorescence (GFP) was used to quantify the level of mitochondrial stress induction. **(B)** Quantification of mitochondrial stress upon feeding worms with increasing concentrations of *dld-1* RNAi bacteria. Data represents the mean ± SD of 8 replicate experiments with ~50 animals per condition. **(C)** An FCCP titration curve was performed in untreated *dld-1(RNAi)* knockdown animals to determine the optimal FCCP concentration for Seahorse analyses. Data represent the mean ± SD of 15 technical replicates per biological replicate, with one animal per condition. **(D)** OCR of wild-type (N2 Bristol) and *dld-1(RNAi)* knockdown animals treated with water (buffer control) or 25 mM DCA, measured by Seahorse assay following sequential addition of mitochondrial function modulators: FCCP (25 µM) and sodium azide (50 mM). Data represent the mean ± SD of 15 technical replicates per biological replicate, with one animal per condition. **(E)** Pyruvate/Lactate ratio in *dld-1*(*1:20* RNAi) knockdown worms trended toward increase relative to wild type (N2 Bristol), and treatment with 25 mM DCA trended to further increase this ratio. *dld-1(1:100* *RNAi*) worms showed no difference in pyruvate/lactate levels compared to wild type, and treatment with DCA did not significantly change this ratio. ****: p<0.0001

**Supplementary Fig. 1.**

**Supplementary Fig. 2.**

**Supplementary Fig. 3**

**Supplementary Fig. 4.**

**Supplementary Fig. 5.**

**Supplementary Fig. 6.**

**Supplementary Fig. 7.**

**Supplementary Fig. 8.**

**Supplementary Fig. 9.**

**Supplementary Fig. 10.**

**Supplementary Fig. 11.**

**Supplementary Fig. 12.**

**Supplementary Table 1.** Comparison of lifespan data, measured using the WormScan method, in presence of FUDR, for *dld-1(RNAi)* knockdown worms from this study and previous reports.

|  | ***wild-type*** | | ***dld-1(RNAi)*** | | ***dld-1***  ***(1:20 RNAi)*** | | ***dld-1(1:100 RNAi)*** | ***pdha-1(RNAi)*** |
| --- | --- | --- | --- | --- | --- | --- | --- | --- |
|  | previous study^1^ (N2 Bristol) | current study  (N2 Bristol) | previous study^1^ | current study | previous study^1^ | current study | current study | current study |
| **Level of protein knockdown determined by western immuno-blotting (relative to wild type)** | N/A | N/A | 71% | 80% | 38% | 56% | 18% | 70% |
| **DCA treatment onset** | L4 | embryo | L4 | embryo | L4 | embryo | embryo | embryo |
| **Median lifespan (days, in water control)** | 21 | 21 | 28 | 14 | 19 | 16 | 16 | 12 |
| **Median lifespan (days, in 25 mM DCA treatment)** | not assessed | 20 | 32 | 19 | 25 | 20 | 16 | 13 |

**Supplementary Table 2.** Overview of the experimental design and conditions presented in this study

| **Experiment** | **Strain** | **Treatment start** | **Measurement time point** |
| --- | --- | --- | --- |
| Lifespan (manual and WormScan assays) | N2 Bristol | embryo | Imaged from stage L4, for 30 days |
| Stimulated locomotor activity measurements (automated light-stimulated activity) | N2 Bristol | embryo | Imaged from stage L4 for 30 days |
| Worm linear growth | N2 Bristol | embryo | L4+1 day |
| Neuromuscular activity (thrashing) | N2 Bristol | embryo | L4+1 day |
| UPR^mt^ stress (high-content imaging) | *hsp-6p::GFP; myo-2p::mCherry* | embryo | L4+1 day |
| Oxygen consumption rate (seahorse analysis) | N2 Bristol | embryo | L4 |
| Mitochondrial membrane potential, mitochondrial mass (Biosorter analysis) | N2 Bristol | embryo | L4+1 day (fluorescent labeling started at stage L4) |
| Measurement of lactate and pyruvate levels | N2 Bristol | embryo | L4+1 day |
| Mitochondrial morphology (confocal microscopy) | SJ4103 strain carrying the zcls14 construct (*myo-3p::GFP(mit)*) | embryo | L4+1 day |

**References**

1.	Broxton, C.N., Kaur, P., Lavorato, M., Ganesh, S., Xiao, R., Mathew, N.D., Nakamaru-Ogiso, E., Anderson, V.E., and Falk, M.J. (2022). Dichloroacetate and thiamine improve survival and mitochondrial stress in a C. elegans model of dihydrolipoamide dehydrogenase deficiency. JCI Insight *7*. 10.1172/jci.insight.156222.
